# Osteogenic-like Phenotypic Reprogramming Is Associated with Reduced Malignant Behaviors in Pancreatic Cancer Cells Involving MAPK–ERK Signaling

**DOI:** 10.3390/ijms27114725

**Published:** 2026-05-24

**Authors:** Gong Chen, Xiaoyan Huang, Dan Li, Weiping Wei

**Affiliations:** 1 Department of General, Visceral & Transplant Surgery, Section Surgical Research, University of Heidelberg, 69120 Heidelberg, Germany; gong.chen@stud.uni-heidelberg.de (G.C.); xiaoyan.huang@uni-ulm.de (X.H.); dan.li@stud.uni-heidelberg.de (D.L.); 2 First Department of Medicine, Medical Faculty Mannheim, University Medical Centre Mannheim (UMM), Heidelberg University, 68167 Mannheim, Germany

**Keywords:** pancreatic ductal adenocarcinoma, phenotypic plasticity, osteogenic-like reprogramming, MAPK–ERK pathway

## Abstract

Pancreatic tumors frequently exhibit calcification, suggesting potential osteogenic-related phenotypic plasticity. This study aimed to systematically evaluate whether pancreatic ductal adenocarcinoma (PDAC) cells acquire osteogenic-like features under induction conditions and to assess the associated phenotypic and molecular changes. PDAC cell lines and non-malignant pancreatic epithelial cells were subjected to osteogenic induction. Mineralization, alkaline phosphatase (ALP) activity, osteogenic marker expression, and malignant phenotypes were evaluated. RNA sequencing was performed at defined time points to characterize transcriptional changes. Pharmacological inhibition of MEK and siRNA-mediated knockdown of RUNX2 were applied to examine the involvement of MAPK–ERK signaling and downstream transcriptional regulation. Osteogenic induction led to calcium deposition and increased ALP activity in a subset of PDAC cell lines, accompanied by upregulation of osteogenic-associated markers, including RUNX2 and SPP1. Induced cells exhibited reduced migration, clonogenicity, invasion, and proliferation. Transcriptomic analysis revealed activation of osteogenesis-related and calcium-transport pathways, along with downregulation of cell cycle programs. MAPK–ERK signaling was activated during induction, and MEK inhibition attenuated RUNX2 and ALP expression as well as mineralization-associated changes. Furthermore, RUNX2 knockdown reduced ALP expression and mineralization levels, indicating its contribution to the osteogenic-like phenotype. PDAC cells can acquire osteogenic-like features under defined induction conditions, accompanied by coordinated transcriptional reprogramming and reduced malignant phenotypes. The observed mineralization-associated phenotypes may reflect a combination of active processes and passive calcium deposition. In addition, the MAPK–ERK–RUNX2 axis appears to be involved in this process, although it may reflect a broader adaptive or stress-associated reprogramming rather than lineage commitment. These findings provide insight into the potential relationship between tumor calcification and phenotypic plasticity in PDAC.

## 1. Introduction

Pancreatic ductal adenocarcinoma (PDAC) is a highly aggressive malignancy characterized by early metastasis, therapeutic resistance, and pronounced cellular heterogeneity [1]. In recent years, increasing attention has been paid to the role of cellular plasticity and transcriptional reprogramming in shaping tumor behavior beyond classical genetic alterations.

Radiological and pathological observations have reported intra- and peritumoral calcification in a subset of pancreatic tumors [2,3,4]. While this phenomenon has been described, its biological basis remains unclear. One possible explanation is that tumor cells may undergo phenotypic changes associated with osteogenic-related programs [5]. However, current evidence is largely limited to descriptive or case-based observations, and whether such features reflect active cellular reprogramming or passive mineral deposition remains unresolved.

Osteogenic-related programs are tightly regulated processes involving coordinated activation of transcription factors such as RUNX2 and downstream effectors including ALP and SPP1, often under the control of signaling pathways such as MAPK–ERK [6,7,8,9,10]. Notably, aberrant expression of osteogenic-associated genes has also been reported in several solid tumors, where it has been variably linked to tumor progression, metastasis, or microenvironmental adaptation [11,12,13]. These observations raise the possibility that osteogenic-related transcriptional programs may be activated outside of classical bone lineage contexts. However, it remains unclear whether PDAC cells can adopt osteogenic-like phenotypes under defined conditions, to what extent such changes represent structured lineage differentiation versus adaptive phenotypic reprogramming, and whether these changes are functionally associated with alterations in malignant behavior.

In this study, we used PDAC cell lines to systematically examine their responses to osteogenic induction conditions. We evaluated mineralization-associated phenotypes, osteogenic marker expression, and changes in malignant characteristics, and performed transcriptomic analysis to characterize the dynamics of gene expression. In addition, we investigated the involvement of MAPK–ERK signaling and the role of RUNX2 using pharmacological inhibition and siRNA-mediated knockdown approaches. Our aim was not to assume a priori lineage commitment, but to characterize the extent and nature of osteogenic-like phenotypic changes in PDAC cells and to explore their potential biological relevance in the context of tumor plasticity and calcification.

## 2. Results

### 2.1. Osteogenic Induction Induces Mineralization-Associated and ALP-Positive Phenotypes in a Subset of PDAC Cell Lines

**Figure 1:**
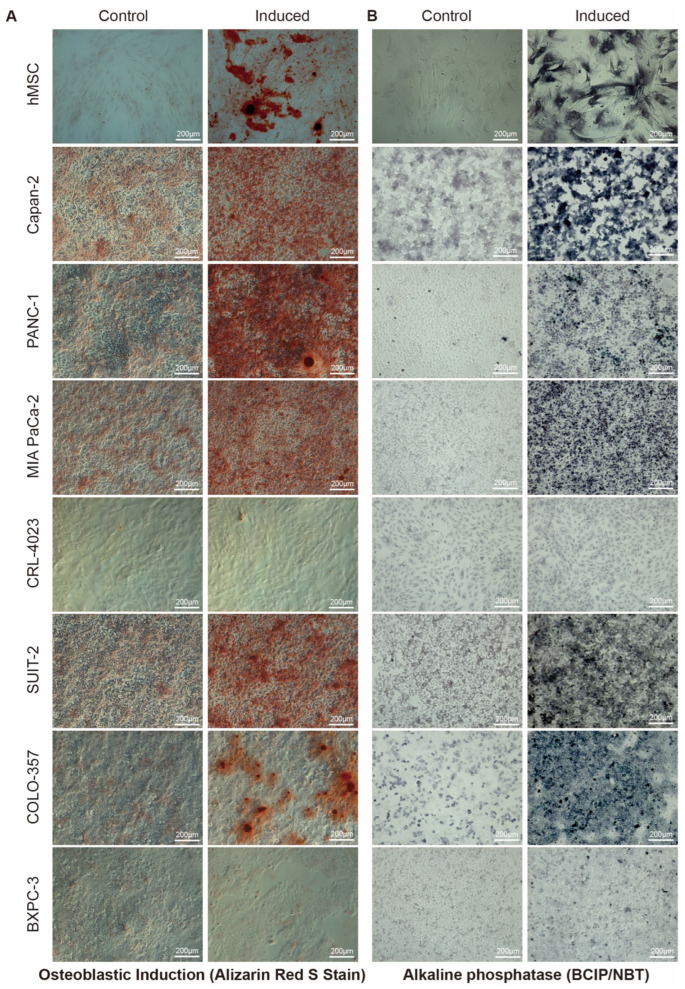
Mineralization and ALP activity of osteogenic-induced PDAC cell line subpopulations. (**A**) Mineralization and ALP activity in human mesenchymal stem cells (hMSCs; positive control), seven pancreatic ductal adenocarcinoma (PDAC) cell lines (Capan-2, PANC-1, MIA PaCa-2, SUIT-2, COLO-357, BXPC-3), and non-malignant pancreatic epithelial cells (CRL-4023) under control and osteogenic induction conditions. Alizarin Red S staining indicates calcium deposition, with positive staining appearing as bright orange-red signals. (**B**) BCIP/NBT staining was performed to assess alkaline phosphatase (ALP) activity in the same cell models. with positive staining visualized as dark blue-violet signals.

To evaluate the response of PDAC cells to osteogenic induction conditions, human PDAC cell lines (Capan-2, PANC-1, MIA PaCa-2, SUIT-2, COLO-357, and BXPC-3) and a non-malignant pancreatic epithelial cell line (CRL-4023) were cultured in osteogenic medium. Calcium deposition was assessed using Alizarin Red S staining, and alkaline phosphatase (ALP) activity was evaluated by BCIP/NBT staining. Human mesenchymal stem cells (hMSCs) were included as a positive control. Induced hMSCs showed apparent mineralization, whereas control hMSCs exhibited minimal staining. Among PDAC cell lines, Capan-2, PANC-1, MIA PaCa-2, SUIT-2, and COLO-357 displayed markedly increased Alizarin Red S staining following induction, with the appearance of dense mineralized regions compared to their respective controls. In contrast, BXPC-3 and CRL-4023 showed minimal staining under both conditions, indicating limited mineral deposition in these models (Figure 1A). Consistently, BCIP/NBT staining revealed increased ALP activity in the same subset of PDAC cell lines following induction, as evidenced by intensified blue-violet precipitation, while control groups remained largely negative. BXPC-3 and CRL-4023 again showed no substantial increase in ALP activity under induction conditions (Figure 1B).

These findings indicate that osteogenic induction conditions promote mineralization-associated and ALP-positive phenotypic changes in a subset of PDAC cell lines, with marked heterogeneity across models.

### 2.2. Osteogenic Induction Is Associated with Increased Expression of ALP and SPP1 in PDAC Cells

**Figure 2:**
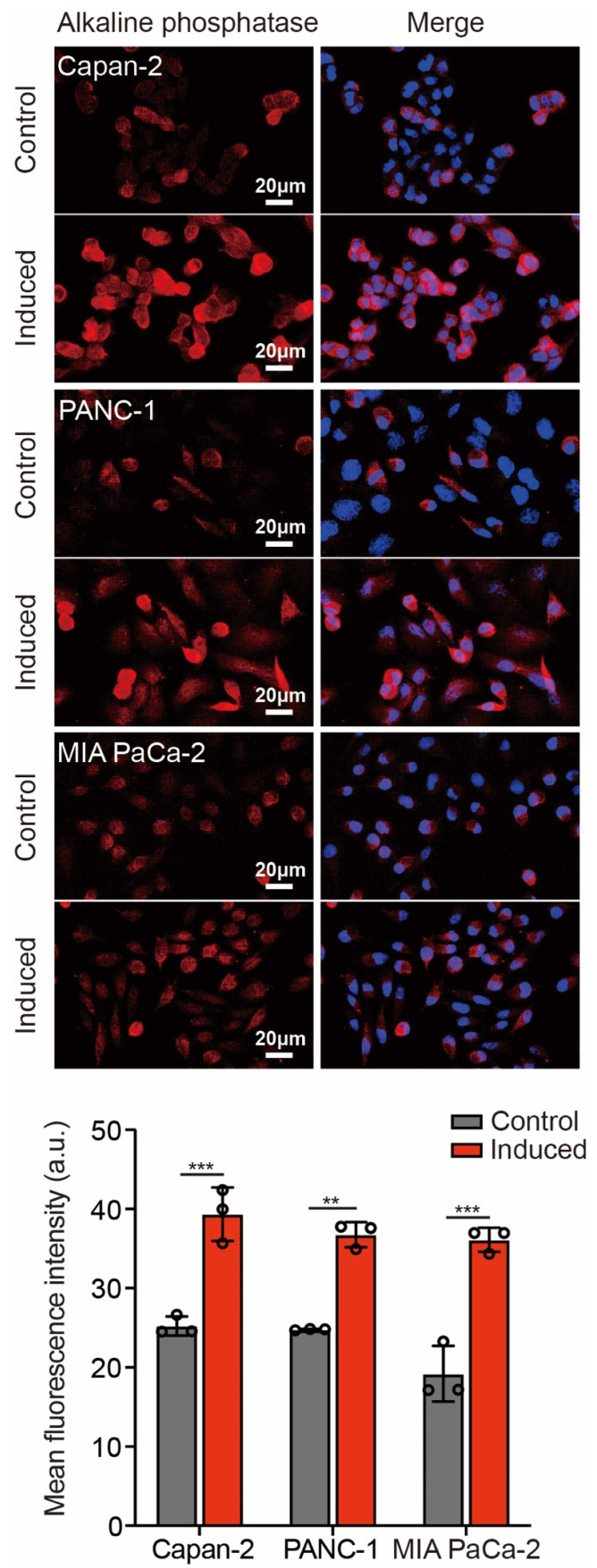
Immunofluorescence detection of ALP expression in PDAC cells after osteogenic induction. Alkaline phosphatase (ALP) immunofluorescence staining of three pancreatic ductal adenocarcinoma (PDAC) cell lines (Capan-2, PANC-1, MIA PaCa-2) under control and osteogenic induction conditions. Quantification of mean fluorescence intensity is shown below. Data are presented as mean ± SD. Statistical significance was determined using Student’s *t*-test (** *p* < 0.01, *** *p* < 0.001).

To further characterize the phenotypic changes observed under osteogenic induction conditions, the expression of osteogenic-associated proteins was evaluated in responsive PDAC cell lines. Immunofluorescence analysis demonstrated that alkaline phosphatase (ALP) expression was markedly increased in induced cells compared to their respective controls after 21 days of treatment. Induced cells exhibited stronger and more uniform cytoplasmic ALP signals, along with an increased proportion of ALP-positive cells within each field (Figure 2). Similarly, the expression of Osteopontin (SPP1) was enhanced following induction, whereas control cells showed weak or negligible staining (Figure S1). The SPP1 signal was predominantly localized in the cytoplasm and perinuclear regions in induced cells. Consistent with these protein-level changes, mRNA expression analysis revealed upregulation of several osteogenic-associated genes, including BGLAP, BMP4, SP7, and RUNX2, in multiple PDAC cell lines (Figure S2A), although the magnitude of change varied across models.

Taken together, these findings indicate that osteogenic induction is associated with coordinated upregulation of osteogenic-associated markers at both the protein and transcriptional levels in a subset of PDAC cells, supporting the activation of an osteogenic-related gene expression program.

### 2.3. Osteogenic Induction Is Associated with Reduced Migration, Clonogenicity, Invasion, and Proliferation in PDAC Cells

**Figure 3:**
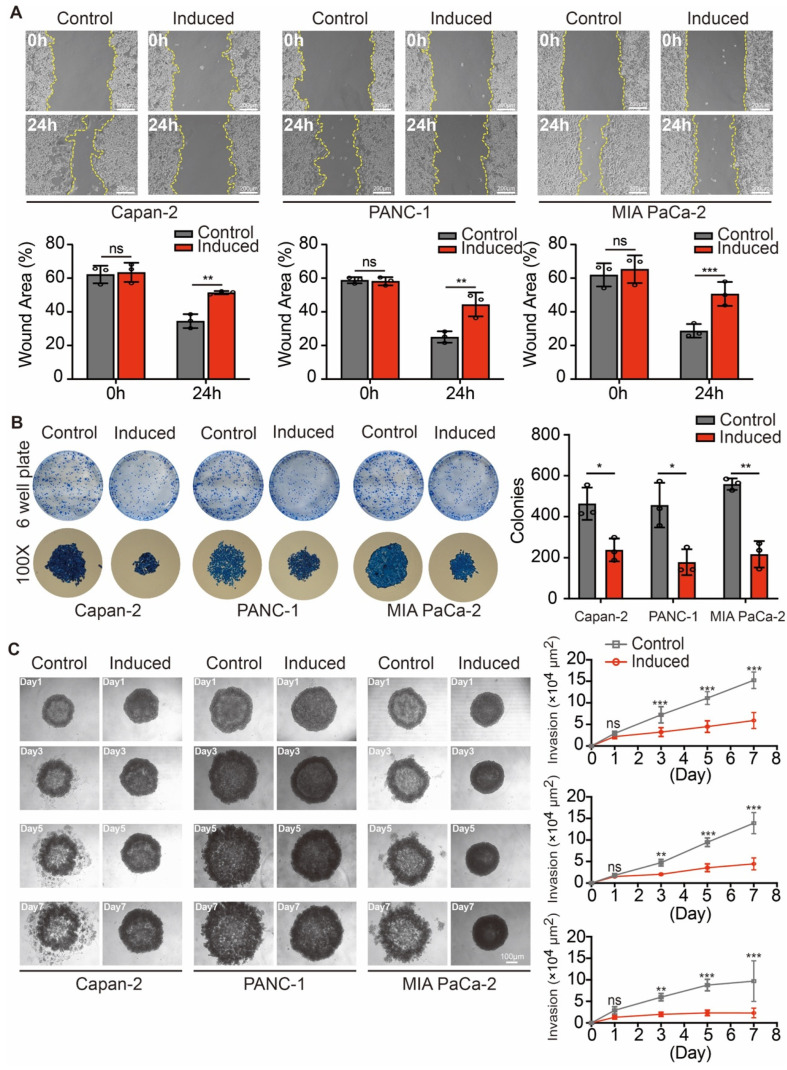
Functional characteristics of PDAC cells after osteogenic induction. (**A**) Wound healing assays of Capan-2, PANC-1, and MIA PaCa-2 cells under control and osteogenic induction conditions. (**B**) Colony formation assays of three PDAC cell lines under control and induced conditions. (**C**) Three-dimensional spheroid invasion assays of Capan-2, PANC-1, and MIA PaCa-2 cells under control and induced conditions. Data are presented as mean ± SD. Statistical significance was determined using Student’s *t*-test (* *p* < 0.05, ** *p* < 0.01, *** *p* < 0.001; ns, not significant).

To assess whether osteogenic induction is accompanied by changes in malignant-associated phenotypes, functional assays were performed in Capan-2, PANC-1, and MIA PaCa-2 cells following 21 days of induction. In wound healing assays, induced cells consistently exhibited reduced migratory capacity compared to control cells, as reflected by a larger residual wound area after 24 h (Figure 3A). Similarly, clonogenic assays demonstrated a marked decrease in both the number and size of colonies formed by induced cells, indicating reduced long-term growth potential (Figure 3B). In the 3D spheroid invasion model, control spheroids displayed progressive outward invasion over time, whereas induced spheroids remained more compact with limited expansion into the surrounding matrix, resulting in significantly smaller invasion areas across all observed time points (Figure 3C). Consistent with these observations, CCK-8 assays showed that induced cells exhibited comparable early growth kinetics but diverged from controls at later time points. By days 5–7, OD_450_ values were consistently lower in induced cells, suggesting a delayed but sustained reduction in proliferative activity (Figure S2B).

Taken together, these results indicate that osteogenic induction conditions are associated with coordinated reductions in multiple malignant-associated cellular behaviors, including migration, clonogenicity, invasion, and sustained proliferation, although these changes may reflect a broader phenotypic shift rather than a specific lineage differentiation process.

### 2.4. Transcriptomic Analysis Reveals Osteogenesis-Associated Transcriptional Reprogramming and Proliferation-Related Gene Suppression During Osteogenic Induction

**Figure 4:**
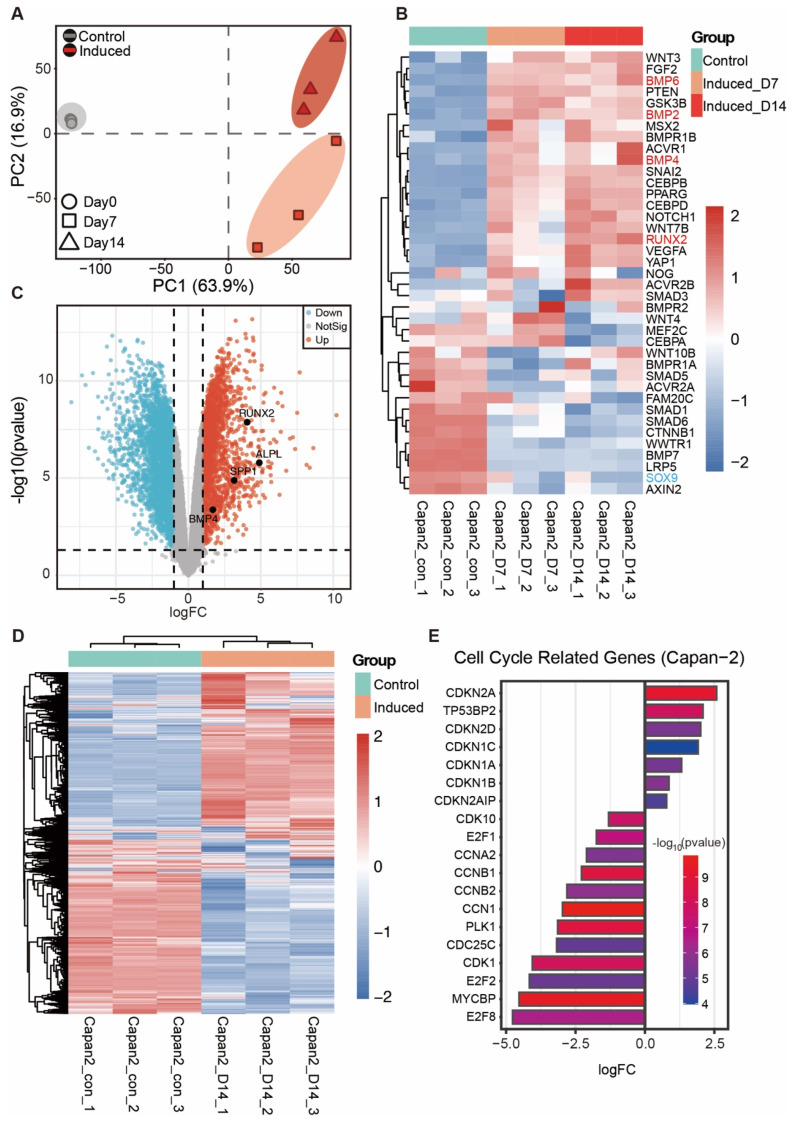
Transcriptomic profiling of PDAC cells after osteogenic induction. (**A**) Principal component analysis (PCA) of Capan-2 cells under control conditions and after 7 days (D7) and 14 days (D14) of osteogenic induction. (**B**) Heatmap of selected osteogenesis- and signaling-related genes in Capan-2 cells during control, D7, and D14 induction stages. (**C**) Volcano plot showing differentially expressed genes in induced Capan-2 cells compared to controls. (**D**) Unsupervised hierarchical clustering heatmap of global transcriptome profiles in control and induced Capan-2 cells. (**E**) Expression changes for cell cycle-related genes in Capan-2 cells.

To systematically characterize molecular changes associated with osteogenic induction, RNA sequencing was performed on Capan-2 and PANC-1 cells at days 7 and 14 following induction. Principal component analysis (Figure 4A) demonstrated clear separation between induced and control samples along PC1, while samples collected at days 7 and 14 were further separated along PC2, indicating progressive transcriptomic divergence over time. Consistently, unsupervised hierarchical clustering of global gene expression profiles showed that induced samples clustered together and were clearly distinct from controls, supporting a time-dependent transcriptional reprogramming process under osteogenic induction conditions. Differential expression analysis identified a substantial number of genes that were significantly upregulated or downregulated after 14 days of induction (Figure 4B). Among the upregulated genes were multiple osteogenesis-associated factors, including RUNX2, BMP4, ALP, and SPP1, which were prominently represented in the upregulated region of the volcano plot (Figure 4C). Notably, these genes showed a progressive increase from day 7 to day 14, suggesting a sustained activation of osteogenesis-related transcriptional programs during induction. Clustering analysis of genes associated with osteogenesis- and chondrogenesis-related processes further supported this trend (Figure 4D). Genes involved in bone-related processes, WNT/BMP signaling, extracellular matrix organization, and calcium ion regulation were upregulated as early as day 7 and exhibited further enhancement at day 14, indicating coordinated activation of multiple osteogenesis-associated gene modules over time. In parallel, a systematic downregulation of cell cycle-related genes was observed (Figure 4E). Key regulators of proliferation, including CDK1, CCNB1/2, E2F family members, and PLK1, showed marked reductions in expression, consistent with suppression of proliferation-associated transcriptional programs during induction.

Together, these data indicate that osteogenic induction is associated with coordinated transcriptional reprogramming in PDAC cells, characterized by activation of osteogenesis-associated gene expression patterns and concurrent downregulation of proliferation-related pathways. Consistent trends were observed in PANC-1 cells (Supplementary Figure S3), where induced samples exhibited clear separation from controls at both day 7 and day 14, with similar patterns of osteogenesis-associated gene upregulation and cell cycle-related gene downregulation. While the magnitude of these changes varied between cell lines, the overall direction of transcriptional responses remained consistent.

### 2.5. MAPK–ERK Signaling Is Activated During Osteogenic Induction and Is Associated with Osteogenesis-Related Gene Expression and Phenotypic Changes in PDAC Cells

**Figure 5:**
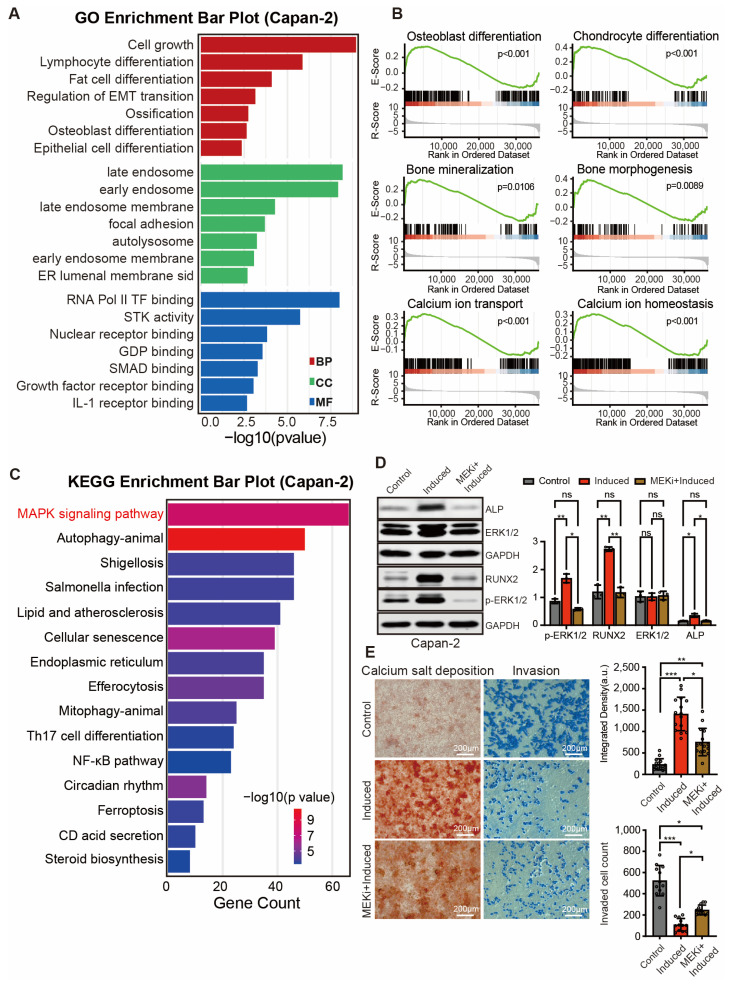
Involvement of MAPK–ERK signaling in osteogenic induction-associated transcriptional and phenotypic changes in PDAC cells. (**A**) Gene Ontology (GO) enrichment analysis of differentially expressed genes in Capan-2 cells under control and osteogenic induction conditions. (**B**) Gene Set Enrichment Analysis (GSEA) plots showing representative gene sets annotated as osteogenesis- and calcium-related pathways in induced Capan-2 cells. (**C**) KEGG pathway enrichment analysis of significantly enriched signaling pathways in induced Capan-2 cells. (**D**) Western blot analysis of ALP, RUNX2, ERK1/2, and phosphorylated ERK1/2 in Capan-2 cells under control, osteogenic induction, and MEK inhibitor (MEKi) treatment conditions. (**E**) Alizarin Red S staining (calcium deposition) and Transwell invasion assays in Capan-2 cells under control, osteogenic induction, and MEKi treatment conditions. Data are presented as mean ± SD. Statistical significance was determined using one-way ANOVA followed by post hoc multiple comparison tests (* *p* < 0.05, ** *p* < 0.01, *** *p* < 0.001; ns, not significant).

GO and KEGG enrichment analyses indicated that osteogenic induction was associated with significant enrichment of biological processes related to bone-associated functions, including gene sets annotated as osteoblast differentiation, cartilage development, bone mineralization, osteogenesis, and calcium ion transport (Figure 5A,C and Figure S4A,C). Concurrently, the MAPK signaling pathway was significantly enriched and exhibited a sustained activation trend from early stages onward (Figure 5B and Figure S4B). GSEA further supported these observations, showing positive enrichment of osteogenesis-related gene sets and coordinated modulation of calcium-associated pathways, suggesting activation of osteogenesis-associated transcriptional programs during induction rather than definitive evidence of lineage differentiation. To further examine the potential involvement of MAPK–ERK signaling, pharmacological inhibition of MEK was applied during osteogenic induction. At the protein level, induction in Capan-2, PANC-1, and MIA PaCa-2 cells was accompanied by increased phosphorylation of ERK, along with elevated expression of RUNX2 and ALP. Upon MEK inhibitor treatment, p-ERK levels were reduced, and the expression of RUNX2 and ALP was correspondingly attenuated (Figure 5D and Figure S4D). At the phenotypic level, osteogenic induction was associated with increased calcium deposition, whereas MEK inhibition reduced mineralization-associated staining intensity (Figure 5E and Figure S4E). In addition, the reduction in invasive capacity observed after induction was partially reversed upon MEK inhibition, as indicated by an increased number of invasive cells compared to the induced group, although not fully restored to control levels.

Together, these findings indicate that MAPK–ERK signaling is activated during osteogenic induction and is associated with modulation of osteogenesis-related gene expression and phenotypic changes in PDAC cells. While these data support a functional involvement of this pathway in the observed reprogramming process, they do not establish a specific or exclusive role as a central driver of lineage differentiation (Figure 5).

### 2.6. RUNX2 Knockdown Attenuates Osteogenesis-Associated Gene Expression and Mineralization-Related Phenotypes in PDAC Cells

**Figure 6:**
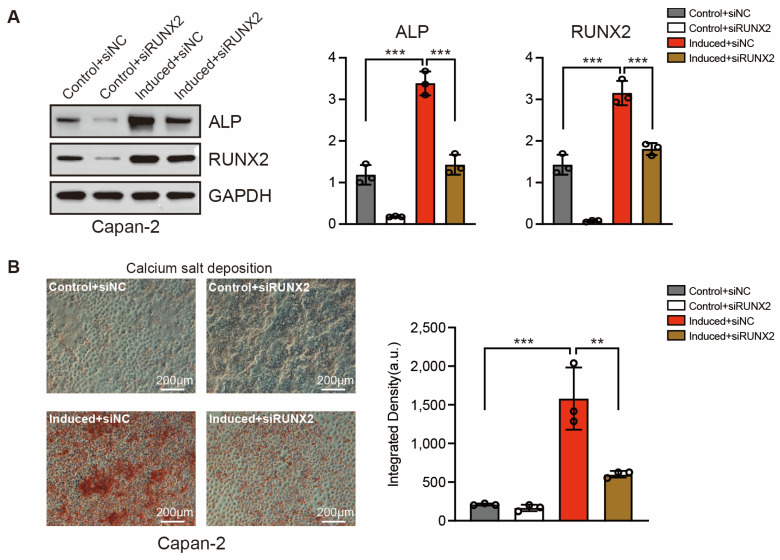
Effects of RUNX2 knockdown on osteogenesis-associated gene expression and mineralization-related phenotypes in PDAC cells. (**A**) Western blot analysis of RUNX2 and ALP expression in Capan-2 cells under control and osteogenic induction conditions with or without siRNA-mediated RUNX2 knockdown (siRUNX2). GAPDH was used as a loading control. (**B**) Alizarin Red S staining of Capan-2 cells under control and osteogenic induction conditions with or without RUNX2 knockdown, showing calcium deposition. Data are presented as mean ± SD. Statistical significance was determined using one-way ANOVA followed by post hoc multiple comparison tests (** *p* < 0.01, *** *p* < 0.001).

To further evaluate the contribution of RUNX2 to the osteogenic induction-associated changes, siRNA-mediated knockdown experiments were performed in Capan-2 cells under control and osteogenic induction conditions. Western blot analysis confirmed that RUNX2 expression was markedly reduced following siRNA transfection in both control and induced groups. Consistently, ALP expression was also decreased upon RUNX2 knockdown, particularly under osteogenic induction conditions (Figure 6A), indicating that RUNX2 is associated with the regulation of osteogenesis-related gene expression. At the phenotypic level, Alizarin Red S staining showed substantial calcium deposition in induced cells, whereas RUNX2 knockdown led to a marked reduction in mineralization-associated staining intensity (Figure 6B). Quantitative analysis further confirmed a significant decrease in mineralization levels in the induced + siRUNX2 group compared to the induced + siNC group.

Taken together, these results indicate that RUNX2 contributes to the osteogenic induction-associated transcriptional and phenotypic changes in PDAC cells. While RUNX2 knockdown attenuates these responses, the findings support a functional role for RUNX2 in this process rather than establishing it as a sole determinant of lineage differentiation.

## 3. Discussion

The most important finding of this study is that, under osteogenic induction conditions, PDAC cells exhibit coordinated mineralization-associated phenotypic changes, activation of osteogenesis-related gene expression programs, and concurrent suppression of malignant-associated behaviors. Importantly, these observations should not be interpreted as evidence of bona fide osteoblastic lineage differentiation. Instead, they more likely reflect an inducible and context-dependent transcriptional and phenotypic reprogramming process, which may partially overlap with osteogenesis-associated programs without constituting true lineage commitment. Previous pathological and radiological studies have reported heterogeneous calcification patterns in pancreatic tumors, although the biological basis of this phenomenon remains unclear [14,15,16,17,18,19,20,21]. One possible explanation is that tumor cells may activate osteogenesis-associated transcriptional programs under specific microenvironmental conditions, resulting in mineral deposition or osteomimicry-like features [22,23,24]. Our in vitro findings provide experimental support for this hypothesis by demonstrating that, under defined induction conditions, PDAC cells can acquire mineralization-associated phenotypes accompanied by coordinated transcriptional changes. Notably, the observed calcium deposition does not definitively demonstrate active matrix mineralization. Under prolonged induction conditions, passive calcium precipitation cannot be excluded. Therefore, the mineralization-associated phenotypes observed in this study should be interpreted with caution and may reflect a combination of active cellular processes and non-specific deposition effects.

Importantly, these osteogenesis-associated responses are not uniformly observed across all PDAC cell lines, indicating marked heterogeneity. Only a subset of PDAC models exhibited mineralization and ALP activity, whereas others, including BXPC-3 and non-malignant epithelial cells, showed minimal responses. This suggests that the capacity to activate osteogenesis-related programs is not solely determined by external stimuli but may depend on intrinsic transcriptional states or epigenetic accessibility [25,26,27]. Such heterogeneity is consistent with the concept of tumor cell plasticity, where only specific subpopulations retain the ability to undergo adaptive transcriptional reprogramming under environmental cues [28]. This is further supported by recent studies demonstrating dynamic state transitions and intercellular signaling networks that maintain phenotypic heterogeneity in PDAC, rather than fixed lineage commitment [29].

Transcriptomic analysis further revealed that osteogenic induction is associated with coordinated upregulation of osteogenesis-related gene modules and downregulation of cell cycle-associated programs. These findings suggest a global shift in cellular state, which is characterized by reduced proliferation and altered transcriptional identity. However, it should be noted that enrichment of gene sets annotated as “osteoblast differentiation” reflects shared transcriptional programs rather than definitive evidence of lineage commitment. Similar transcriptional patterns have been described in the context of tumor cell plasticity and osteomimicry in other solid tumors [12,30,31].

Functionally, osteogenic induction was associated with reduced migration, clonogenicity, invasion, and sustained proliferation. While these changes may resemble a “less aggressive” cellular phenotype, they do not necessarily indicate terminal differentiation. An alternative explanation is that these phenotypic alterations may reflect stress-associated growth restriction, metabolic adaptation, or transcriptional remodeling induced by the non-physiological culture conditions [32]. Additionally, the osteogenic induction medium contains potent non-physiological biochemical components, including dexamethasone, β-glycerophosphate, and ascorbic acid. The metabolic and signaling perturbations induced by these agents may also contribute to the observed transcriptional and phenotypic alterations, rather than exclusively reflecting specific lineage-directed osteogenic programming.

Our data indicate that MAPK–ERK signaling is activated during osteogenic induction and is associated with osteogenesis-related gene expression and phenotypic changes. Pharmacological inhibition of MEK reduced RUNX2 and ALP expression and attenuated mineralization-associated phenotypes, suggesting functional involvement of this pathway. However, given the pleiotropic roles of ERK signaling in proliferation, stress response, and differentiation [33,34], these results do not establish MAPK–ERK as a specific or exclusive driver of osteogenic reprogramming. Instead, ERK activation may represent a context-dependent signaling node whose downstream outputs are shaped by transcriptional and environmental conditions. Consistent with previous studies, signaling pathways associated with MAPK and PP2A have been shown to regulate multiple fundamental cellular processes in PDAC, including proliferation, apoptosis, and adaptive stress responses, rather than functioning as lineage-specific determinants [35]. Furthermore, recent evidence suggests that such signaling networks can govern adaptive cellular states, including apoptotic dormancy and therapy resistance, highlighting their role in dynamic and reversible cell state regulation [36].

RUNX2, a key transcription factor in bone biology [37], has also been implicated in tumor progression, metastasis, and metabolic regulation across multiple cancer types [38,39]. In this study, RUNX2 knockdown attenuated ALP expression and reduced mineralization-associated phenotypes, supporting its functional contribution to osteogenesis-associated transcriptional programs in PDAC cells. Nevertheless, these findings do not establish RUNX2 as a master regulator of lineage differentiation in this context, but rather as one component of a broader regulatory network involved in transcriptional reprogramming.

The findings of this study align with the concept of phenotypic plasticity in cancer, where tumor cells can temporarily adopt alternative transcriptional states in response to environmental cues. In bone metastasis models, similar activation of osteogenic-like programs—often referred to as osteomimicry—has been linked to tumor adaptation and colonization within the bone microenvironment. In contrast, in the present in vitro context, activation of osteogenesis-associated programs is accompanied by reduced malignant-associated behaviors, highlighting the context-dependent functional consequences of such reprogramming.

These observations also raise the possibility that osteogenic-like reprogramming may contribute to tumor calcification in PDAC. Rather than representing purely passive mineral deposition, calcification may, in part, reflect active cellular processes associated with transcriptional state changes. However, this hypothesis remains speculative and requires validation in clinical specimens, including spatial and molecular correlation analyses between calcification and tumor cell phenotypes.

Several limitations should be acknowledged. First, the osteogenic induction system used in this study is based on a non-physiological combination of biochemical factors, which may introduce metabolic stress or non-specific effects. Second, the absence of in vivo or organoid-based validation limits the translational interpretation of these findings. Third, the reversibility and stability of the osteogenesis-associated state remain unclear, and it is not known whether these changes persist after withdrawal of induction conditions. Addressing these questions will require future studies integrating single-cell transcriptomics, epigenetic profiling, and lineage tracing approaches.

In summary, this study demonstrates that PDAC cells can undergo osteogenesis-associated transcriptional and phenotypic reprogramming under defined induction conditions, accompanied by reduced malignant-associated behaviors. The MAPK–ERK–RUNX2 axis appears to be involved in this process, although its role is likely context-dependent and not restricted to lineage specification. These findings provide a conceptual framework linking tumor calcification, transcriptional plasticity, and phenotypic state transitions in PDAC, and may inform future investigations into differentiation-associated therapeutic strategies.

## 4. Materials and Methods

### 4.1. Cell Lines and Culture

Human pancreatic ductal adenocarcinoma (PDAC) cell lines Capan-2, PANC-1, MIA PaCa-2, SUIT-2, COLO-357, and BxPC-3 and the non-malignant human pancreatic duct epithelial cell line CRL-4023 were purchased from the American Type Culture Collection (ATCC, Manassas, VA, USA). Human bone marrow-derived mesenchymal stem cells (hMSCs) were previously isolated and stored by our research group and served as a positive control for osteogenic induction. All cells were cultured in DMEM supplemented with 10% FBS under standard conditions at 37 °C and 5% CO_2_ with saturated humidity. Routine morphological observation and the PlasmoTest™ kit (InvivoGen, San Diego, CA, USA) were used to screen for mycoplasma contamination, and cell line identification was confirmed via SNP genotyping.

### 4.2. Osteogenic Induction

Cells were seeded in 6-well plates. Upon reaching approximately 2–3 × 10^5^ cells per well, cells were cultured in commercial osteogenic induction medium (NH OsteoDiff (Miltenyi Biotec, Bergisch Gladbach, Germany) as the induced group, this medium contains a defined combination of osteogenesis-associated supplements, including dexamethasone, β-glycerophosphate, and ascorbic acid, which are commonly used to promote mineralization-associated phenotypes in vitro [40]. Control group cells were maintained in conventional medium. The medium was refreshed every 2–3 days. Cells were harvested on day 21 for Alizarin Red S staining, BCIP/NBT staining, immunofluorescence, qRT-PCR, Western blot analysis, and functional assays, including wound healing, colony formation, 3D spheroid invasion, Transwell invasion, and CCK-8 proliferation assays. For transcriptomic analysis, cells were collected on days 7 and 14.

### 4.3. Alizarin Red S Staining

Alizarin Red S staining was employed to detect extracellular calcium salt deposition after induction [41]. Cells were washed twice with PBS, fixed with 4% paraformaldehyde for 15 min, rinsed three times with deionized water, then incubated with Alizarin Red S working solution (Sigma-Aldrich, St. Louis, MO, USA) at room temperature in the dark for 20 min. The staining solution was discarded, and the slides were repeatedly rinsed with deionized water until the background was clear. Images were captured under an inverted microscope.

### 4.4. BCIP/NBT Alkaline Phosphatase Staining

BCIP/NBT reagents are used to detect cellular alkaline phosphatase activity [42]. Cells were fixed for 10 min at 4 °C with pre-cooled 70% ethanol. BCIP/NBT working solution (VECTOR Laboratories, Newark, CA, USA) was prepared according to the kit instructions and incubated at room temperature in the dark for 30–45 min. When a distinct blue-violet insoluble precipitate appeared, the reaction was terminated with deionized water, and images were immediately captured under an inverted microscope.

### 4.5. RNA Extraction and Real-Time Quantitative PCR

Total RNA was extracted using the RNeasy Mini Kit. cDNA was synthesized using the High-Capacity cDNA Reverse Transcription Kit. qRT-PCR was performed on the StepOnePlus real-time PCR system with PowerUp SYBR Green Master Mix. The target genes included BGLAP, BMP4, SP7, and RUNX2. The primers (Eurofins Genomics, Ebersberg, Germany) used are listed in Table 1. Relative expression levels were calculated using the 2^−ΔΔCt^ method [43].

### 4.6. Immunofluorescence Staining

Cells were seeded onto coverslips. After 21 days of induction, the cells were washed with PBS, fixed with 4% paraformaldehyde for 15 min, permeabilized with 0.2% Triton X-100 for 10 min, and blocked with 5% bovine serum albumin (BSA) at room temperature for 1 h. Anti-ALP or anti-Osteopontin (SPP1) primary antibodies were added and incubated overnight at 4 °C. Alexa Fluor 594- or 488-labeled goat anti-rabbit secondary antibody (1:400) was added and incubated at room temperature in the dark for 1 h. Nuclei were stained with DAPI for 5 min, and images were acquired using a fluorescence microscope and the TissueFAXS system. The average fluorescence intensity was quantified using ImageJ software (version 1.54g). The antibodies used are listed in Table 2.

### 4.7. Wound Healing Assay

Cells were seeded in 6-well plates. When the cells had reached approximately 90% confluence, linear scratches were produced using a pipette tip. Photographs were taken at 0 h and 24 h. Image J was used to calculate the remaining wound area, expressed as a percentage of the initial area.

### 4.8. Clonogenic Assay

After 21 days of induction, cells were digested and resuspended in routine culture medium. Then, they were seeded in a 6-well plate (700 cells/well) and cultured in DMEM supplemented with 10% FBS for 10–14 days. The cells were fixed with 4% paraformaldehyde for 20 min, stained with 0.1% crystal violet for 20 min, washed with PBS, and air-dried. ImageJ was used to count the colonies per well, defining each cluster containing ≥50 cells as one colony.

### 4.9. Cell Proliferation Assay (CCK-8)

Cells were re-seeded into 96-well plates (2000 cells/well) after 21 days of induction, with 5 replicate wells per group. On days 1, 3, 5, and 7 post-seeding, 10 µL of CCK-8 working solution was added to each well. After incubation at 37 °C for 2 h, absorbance was measured at 450 nm, and proliferation curves were plotted.

### 4.10. Three-Dimensional Spheroid Invasion Assay

The 3D invasion assay was performed using 96-well plates with ultra-low-binding U-bottom wells. After 21 days of culture under osteogenic induction or control conditions, cells were seeded at a density of 5 × 10^3^ cells/well in DMEM containing 2.5% FBS and cultured for 48 h to form compact cell spheroids. Pre-cooled Matrigel was then added to achieve a final concentration of 5%. Cultures were maintained, with images captured under an inverted microscope on days 1, 3, 5, and 7. The invasion area was measured using Image J.

### 4.11. Western Blot Analysis

Cells were collected and cultured for 21 days under control, osteogenic induction, and “MEK inhibition + osteogenic induction” conditions. Total proteins were extracted using RIPA lysis buffer and quantified via the BCA method. Proteins (20–40 µg per well) were separated via SDS-PAGE and transferred to PVDF membranes. After blocking with 5% BSA at room temperature for 1 h, primary antibodies were incubated overnight at 4 °C. The next day, they were incubated with IRDye-labeled secondary antibody for 1 h. Bands were detected on the Odyssey CLx imaging system, and grayscale quantification was performed using the ImageJ software (version 1.54g) with quantities normalized to that of GAPDH. The antibodies used are listed in Table 2.

### 4.12. Transwell Invasion Assay and MEK Inhibition

The upper chamber was coated with 50 µg/mL Matrigel and allowed to solidify at 37 °C for 2 h; an 8 µm pore size was used. Cells were digested and counted after 21 days of control treatment, osteogenic induction, or osteogenic induction combined with the MEK inhibitor PD98059. The cells were resuspended in serum-free medium and seeded into the upper chamber (6 × 10^4^ cells/chamber), while the lower chamber was filled with medium containing 20% FBS as a chemotactic factor. After 48 h of incubation at 37 °C, the cells were fixed with 4% paraformaldehyde and stained with crystal violet for 20 min, non-migrated cells in the upper chamber were removed with a cotton swab. Three random fields were counted for invasive cells, and the average was calculated.

### 4.13. RNA Sequencing and Bioinformatics Analysis

Total RNA was extracted from Capan-2 and PANC-1 cells cultured under control, 7-day, and 14-day osteogenic induction conditions. Libraries were prepared using the Illumina Stranded mRNA Prep Kit and sequenced using paired-end reads on the NovaSeq 6000 platform. Raw data quality was assessed using FastQC (v0.11.9) and MultiQC (v1.14). Trim Galore was employed for adapter removal and low-quality sequence trimming. Alignment to the human reference genome GRCh38 was performed using STAR (v2.7.10a). Gene-level expression quantification was conducted with featureCounts (subread v2.0.3), and differential expression analysis was completed using the limma package (R v4.2.1), (threshold |log_2_FC| > 1, FDR < 0.05). clusterProfiler was used to perform GO and KEGG enrichment analysis, while fgsea was used to conduct gene set enrichment analysis (GSEA). The results were visualized using ggplot2 (v3.4.2) and enrichplot (v1.18.0).

### 4.14. Small Interfering RNA Transfection

RUNX2 expression was silenced using small interfering RNA (siRNA). Cells were transfected with RUNX2-specific siRNA (siRUNX2; OriGene, Rockville, MD, USA; SR319570) or negative control siRNA (siNC) using Lipofectamine 3000 (Invitrogen, Waltham, MA, USA) according to the manufacturer’s instructions. After transfection, cells were cultured under control or osteogenic induction conditions as described above. Knockdown efficiency was confirmed by Western blot analysis. Subsequent experiments were performed 48–72 h after transfection.

### 4.15. Statistical Analysis

All experiments were performed with at least three independent biological replicates, and data are presented as the mean ± SD. Statistical analyses were conducted using the GraphPad Prism 9.0 and R software (version 4.2.1). Comparisons between two groups were performed using an unpaired two-tailed Student’s t-test, while multiple group comparisons were analyzed by means of one-way ANOVA followed by Bonferroni post hoc tests. * *p* < 0.05; ** *p* < 0.01; *** *p* < 0.001; ns indicates no significant difference.

## 5. Conclusions

This study demonstrates that pancreatic ductal adenocarcinoma cells can acquire osteogenesis-associated phenotypic features under defined induction conditions, which is accompanied by transcriptional reprogramming and reduced malignant-associated behaviors. These changes include mineralization-associated phenotypes, activation of osteogenesis-related gene expression programs, and suppression of proliferation, migration, and invasion.

Importantly, these findings do not indicate definitive lineage differentiation, but rather support the existence of a context-dependent and inducible phenotypic reprogramming process in a subset of PDAC cells. The observed heterogeneity among different cell lines further suggests that only specific subpopulations retain the capacity to activate osteogenesis-associated transcriptional programs, that potentially depending on intrinsic transcriptional states or epigenetic accessibility.

Mechanistically, the MAPK–ERK–RUNX2 axis appears to be involved in this process and contributes to the regulation of osteogenesis-associated gene expression and phenotypic changes. However, this signaling network likely functions as part of a broader context-dependent regulatory system rather than as a specific driver of lineage commitment.

Overall, this study provides a conceptual framework linking tumor calcification, transcriptional plasticity, and phenotypic state transitions in PDAC; however, the biological and clinical significance of these observations remains to be established in future studies.

## Figures and Tables

**Table 1:** Primers used in this study.

Gene Names	Primers	Sequences (5′-3′)
BGLAP	Forward Reverse	CACTCCTCGCCCTATTGGC CCCTCCTGCTTGGACACAAAG
BMP4	Forward Reverse	ATGATTCCTGGTAACCGAATGC CCCCGTCTCAGGTATCAAACT
SP7	Forward Reverse	CCTCTGCGGGACTCAACAAC AGCCCATTAGTGCTTGTAAAGG
RUNX2	Forward Reverse	TGGTTACTGTCATGGCGGGTA TCTCAGATCGTTGAACCTTGCTA
GAPDH	Forward Reverse	GGAGCGAGATCCCTCCAAAAT GGCTGTTGTCATACTTCTCATGG

**Table 2:** Antibodies used in this study.

Antibody	Source	Cat#	Dilution
Alkaline phosphatase (ALP)	Abcam	ab65834	IF 1:500; WB 1:1000
Osteopontin (SPP1)	Abcam	ab63856	1:500
RUNX2	Abcam	ab76956	1:200
ERK1/2	Cell Signaling	9102	1:1000
phosphorylated ERK1/2	Cell Signaling	4370	1:2000
GAPDH	Cell Signaling	2118	1:1000

## Data Availability

The data generated in this study are publicly available in Gene Expression Omnibus (GEO) at GSE314240. The data generated in this study are available within the article and its supplementary data files.

## Supplementary Materials

The following supporting information can be downloaded at: https://www.mdpi.com/article/10.3390/ijms27114725/s1.

## Abbreviations

The following abbreviations are used in this manuscript:

Abbreviation	Full Name
PDAC	Pancreatic ductal adenocarcinoma
ALP	Alkaline phosphatase
ALPL	Alkaline phosphatase, liver/bone/kidney
SPP1	Secreted phosphoprotein 1 (osteopontin)
RUNX2	Runt-related transcription factor 2
BGLAP	Bone gamma-carboxyglutamate protein (osteocalcin)
BMP4	Bone morphogenetic protein 4
SP7	Sp7 transcription factor (osterix)
MAPK	Mitogen-activated protein kinase
ERK	Extracellular signal-regulated kinase
MEK	MAPK/ERK kinase
GSEA	Gene set enrichment analysis
GO	Gene ontology
KEGG	Kyoto Encyclopedia of Genes and Genomes
DEG	Differentially expressed genes
qRT-PCR	Quantitative reverse transcription polymerase chain reaction
CCK-8	Cell Counting Kit-8
hMSCs	Human mesenchymal stem cells
ATCC	American Type Culture Collection
