# Supplementary figures and images for: Osteogenic-like Phenotypic Reprogramming Is Associated with Reduced Malignant Behaviors in Pancreatic Cancer Cells Involving MAPK–ERK Signaling
Source: Int J Mol Sci. 2026 May 24;27(11):4725. doi: 10.3390/ijms27114725 (PMC13256410; doi:10.3390/ijms27114725)

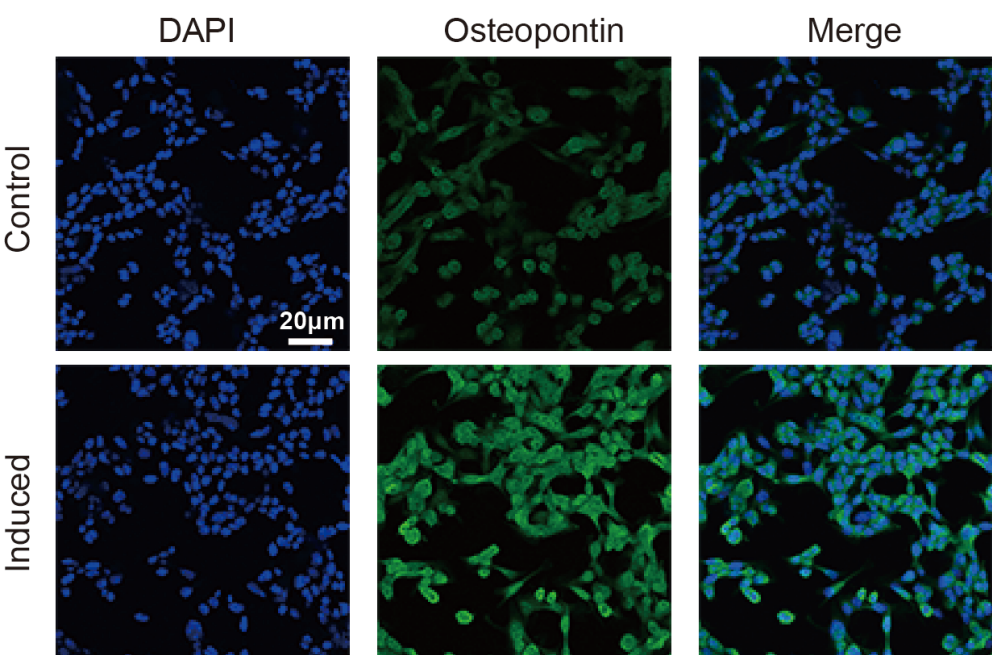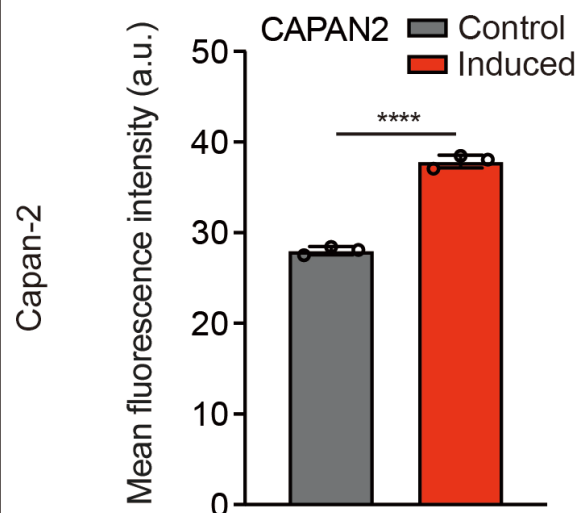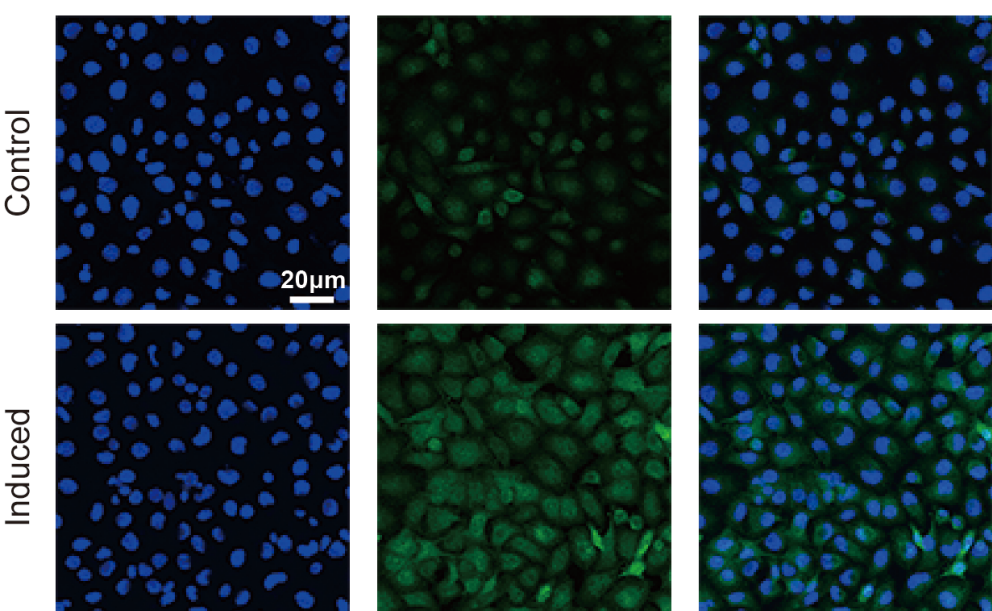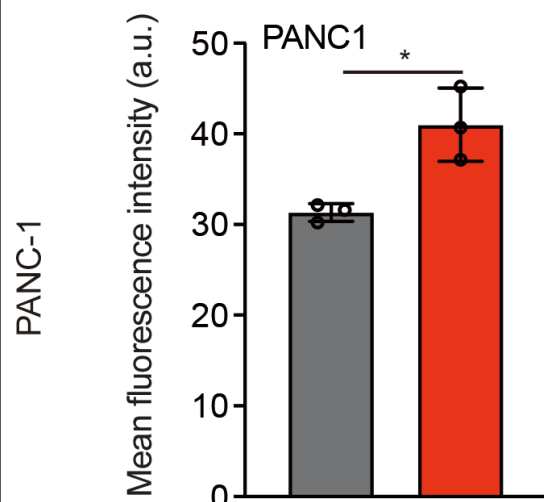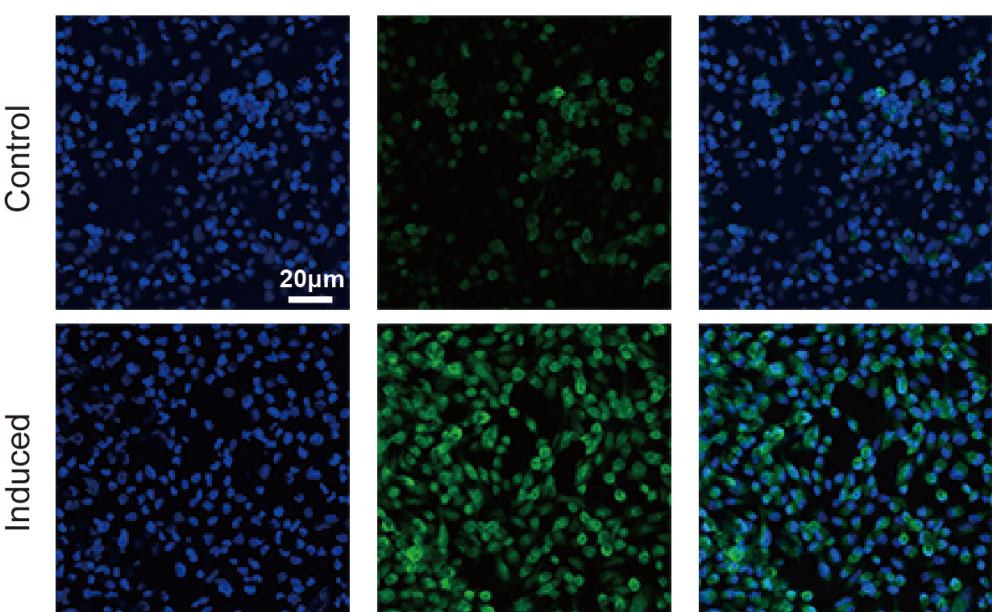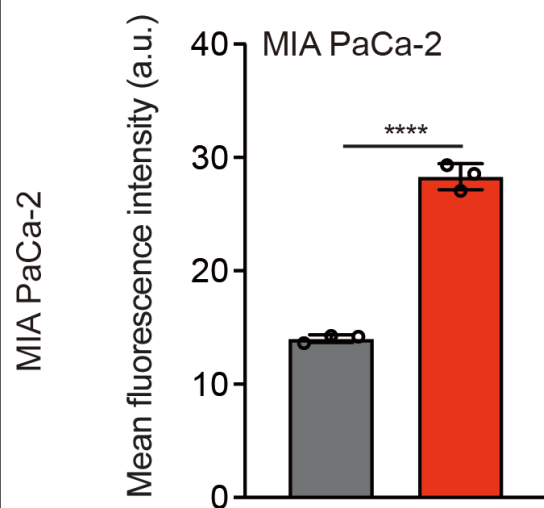

Supplement: Supplementary file 1 [file ijms-27-04725-s001.zip › FigureS1.pdf]

**A**

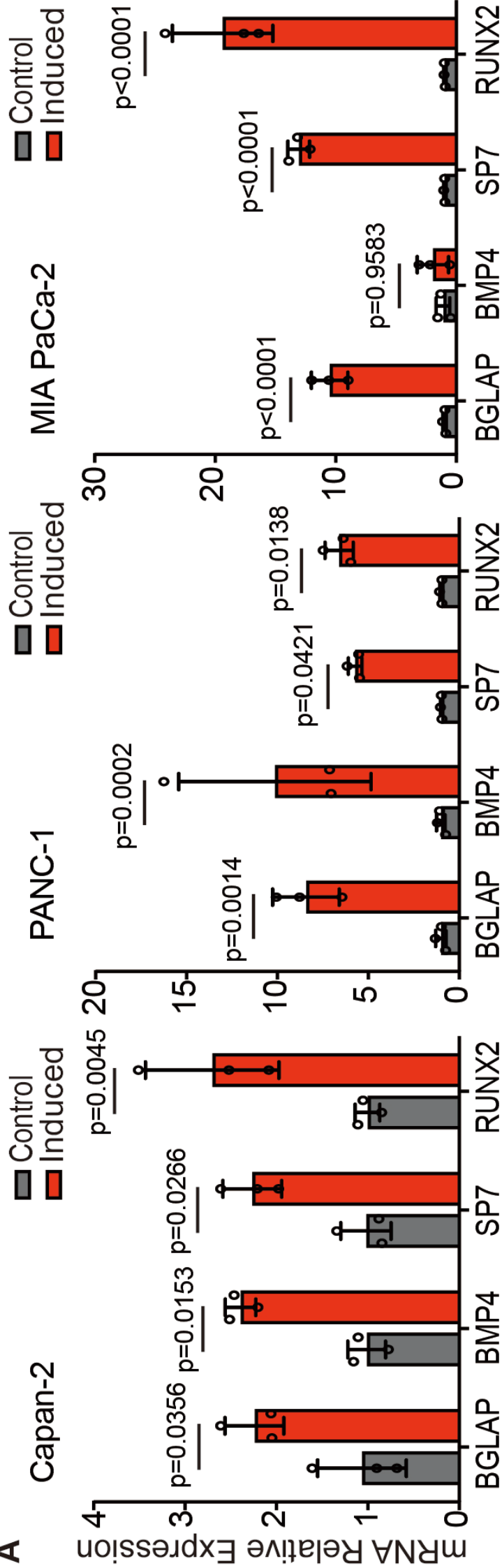

**B**

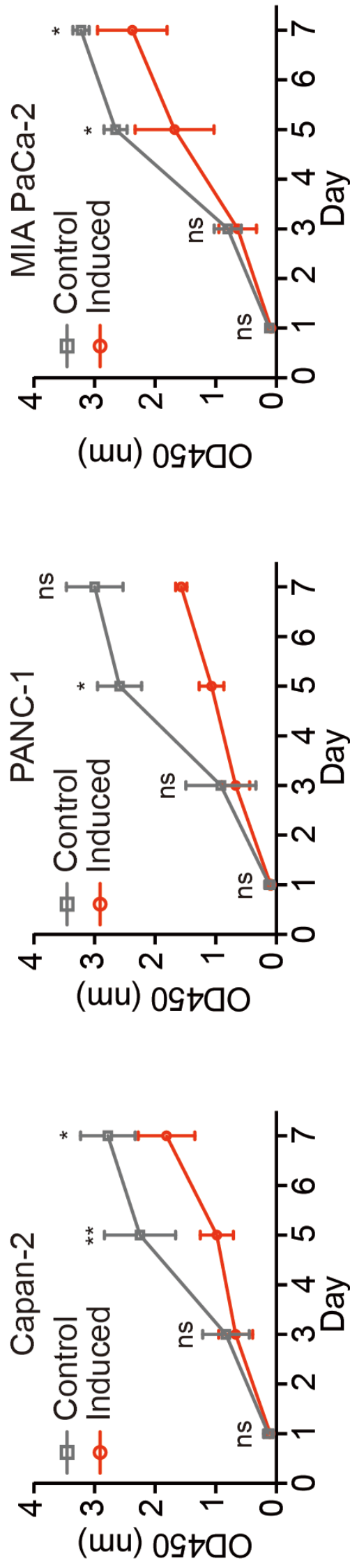

Supplement: Supplementary file 1 [file ijms-27-04725-s001.zip › FigureS2.pdf]

**A**

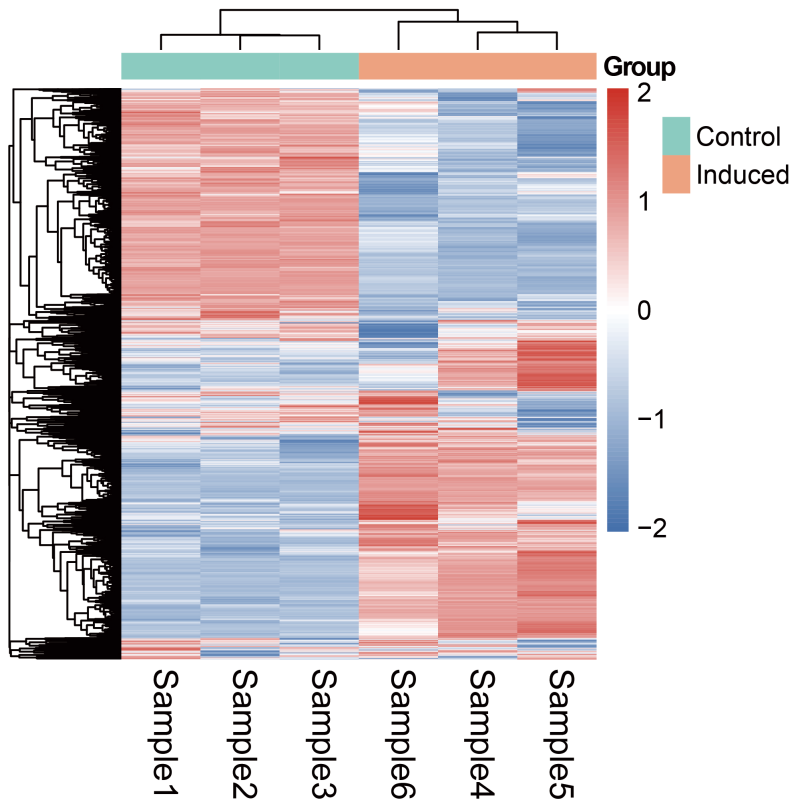

**B**

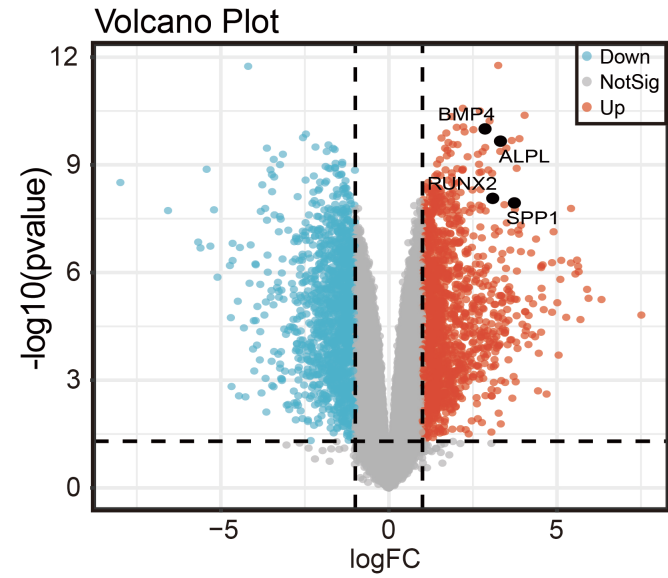

**C**

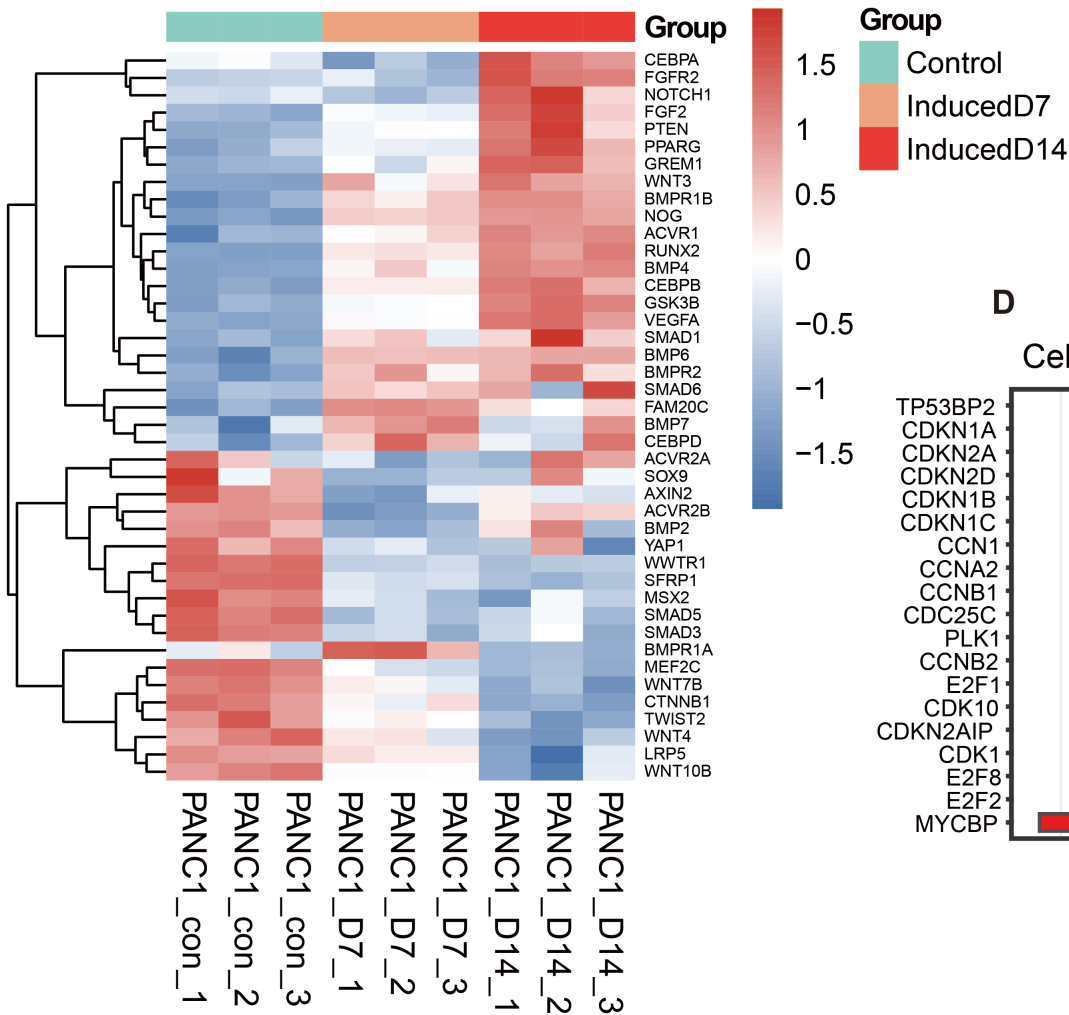

**D**

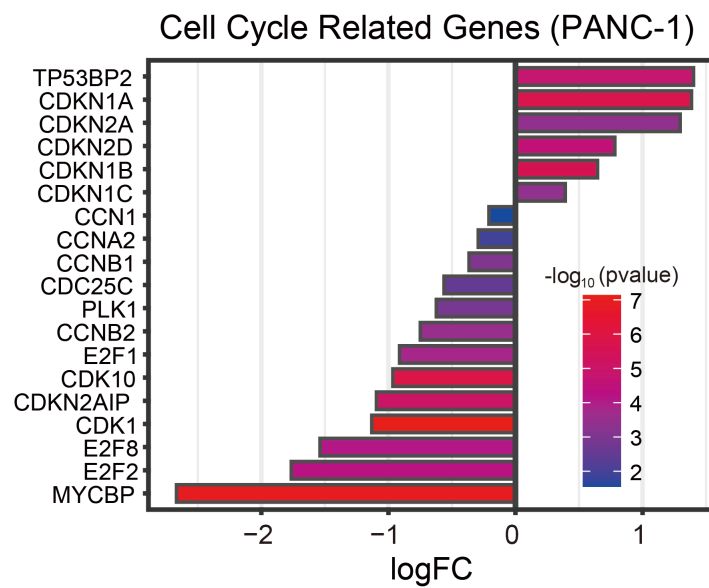

Supplement: Supplementary file 1 [file ijms-27-04725-s001.zip › FigureS3.pdf]

A

## GO Enrichment Bar Plot(PANC-1)

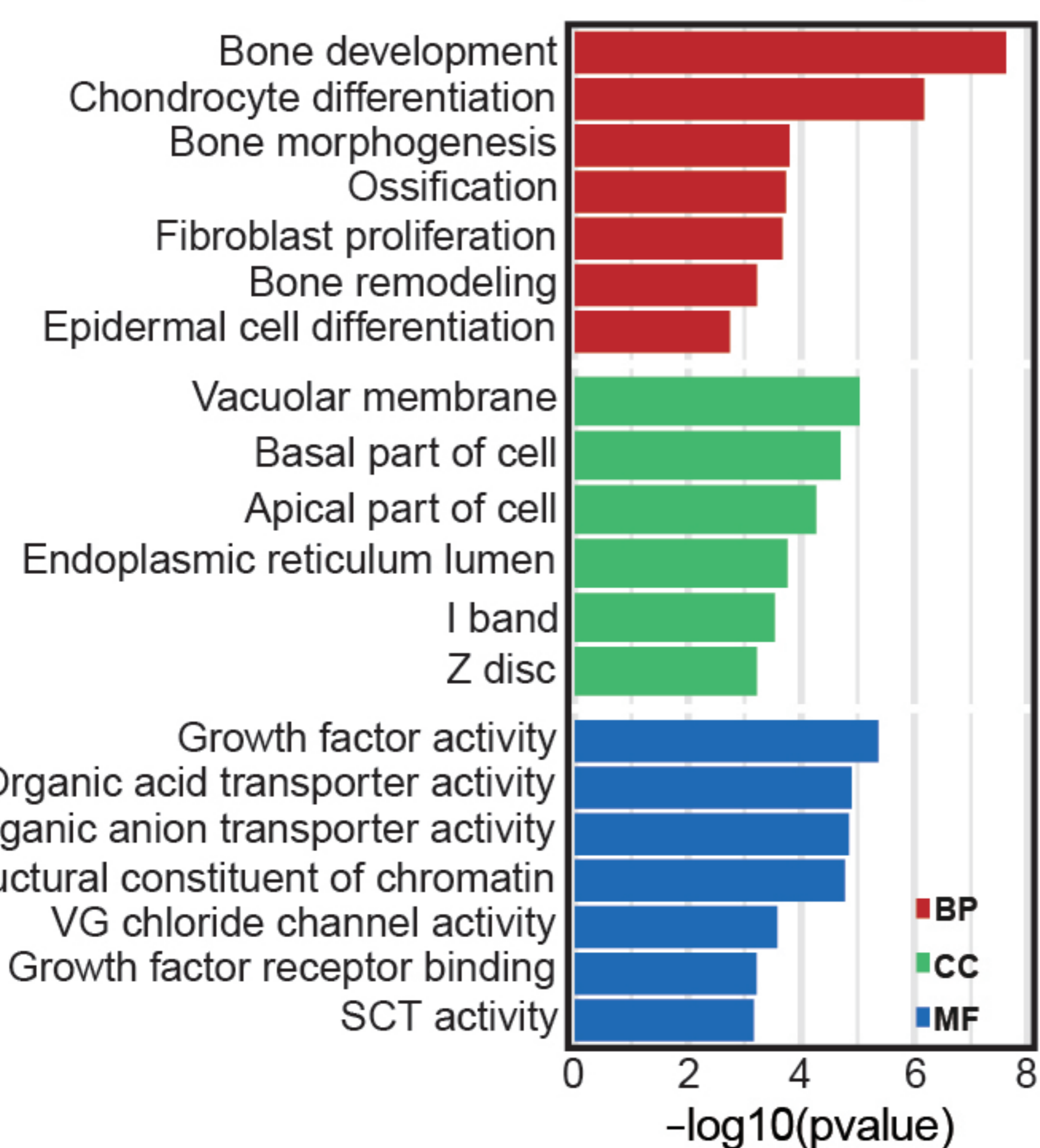

B

## KEGG Enrichment Bar Plot (PANC-1)

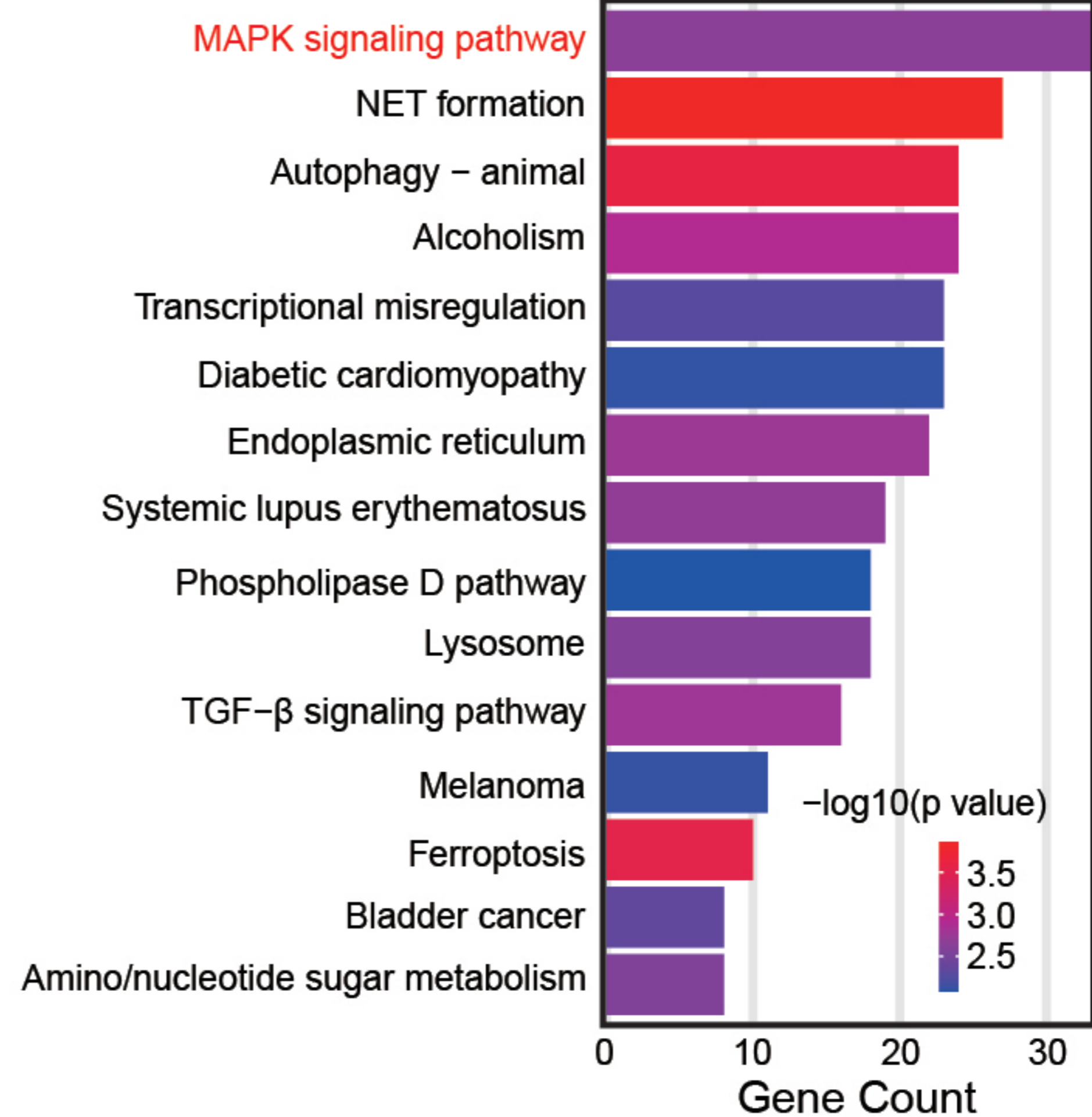

C

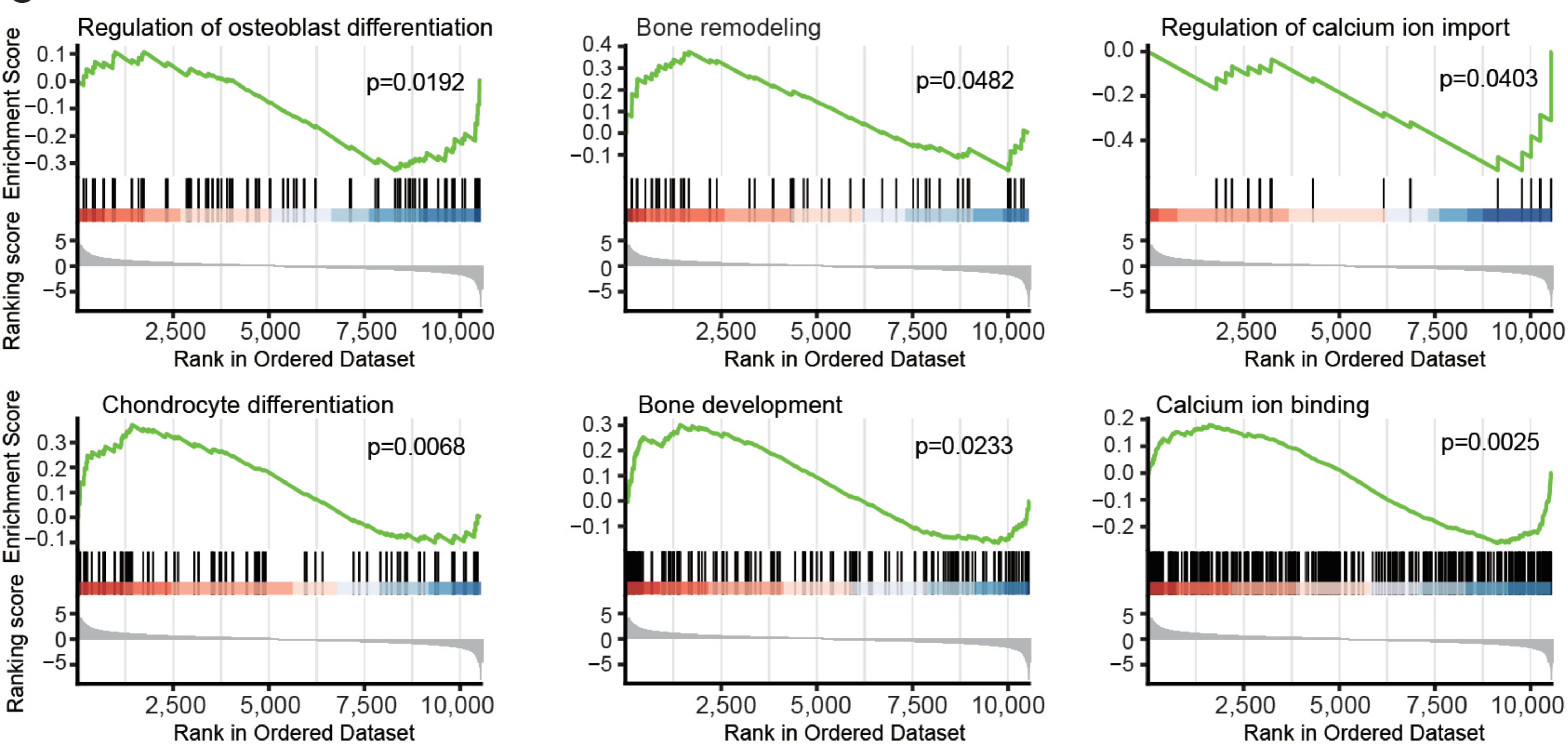

D

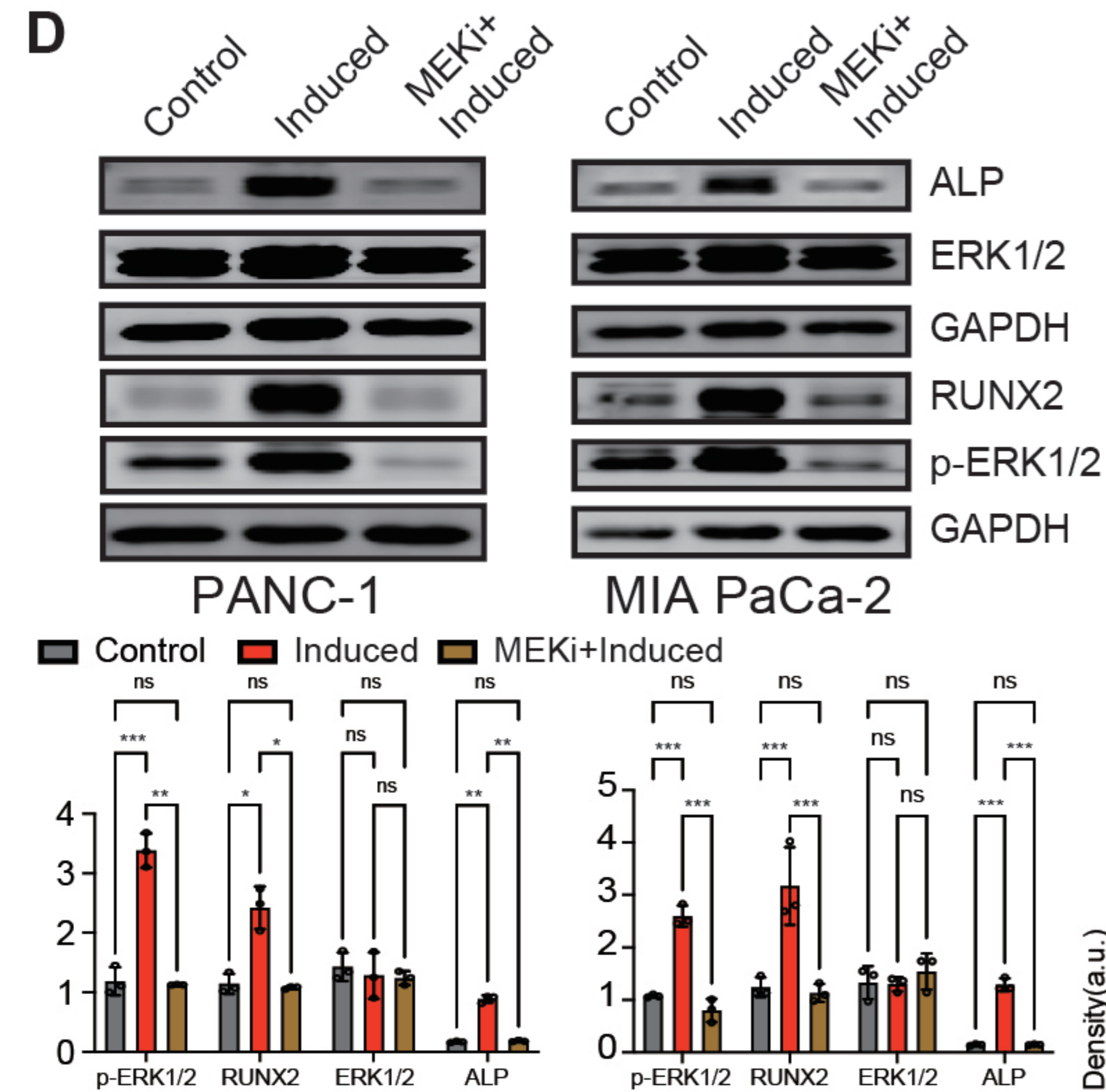

E

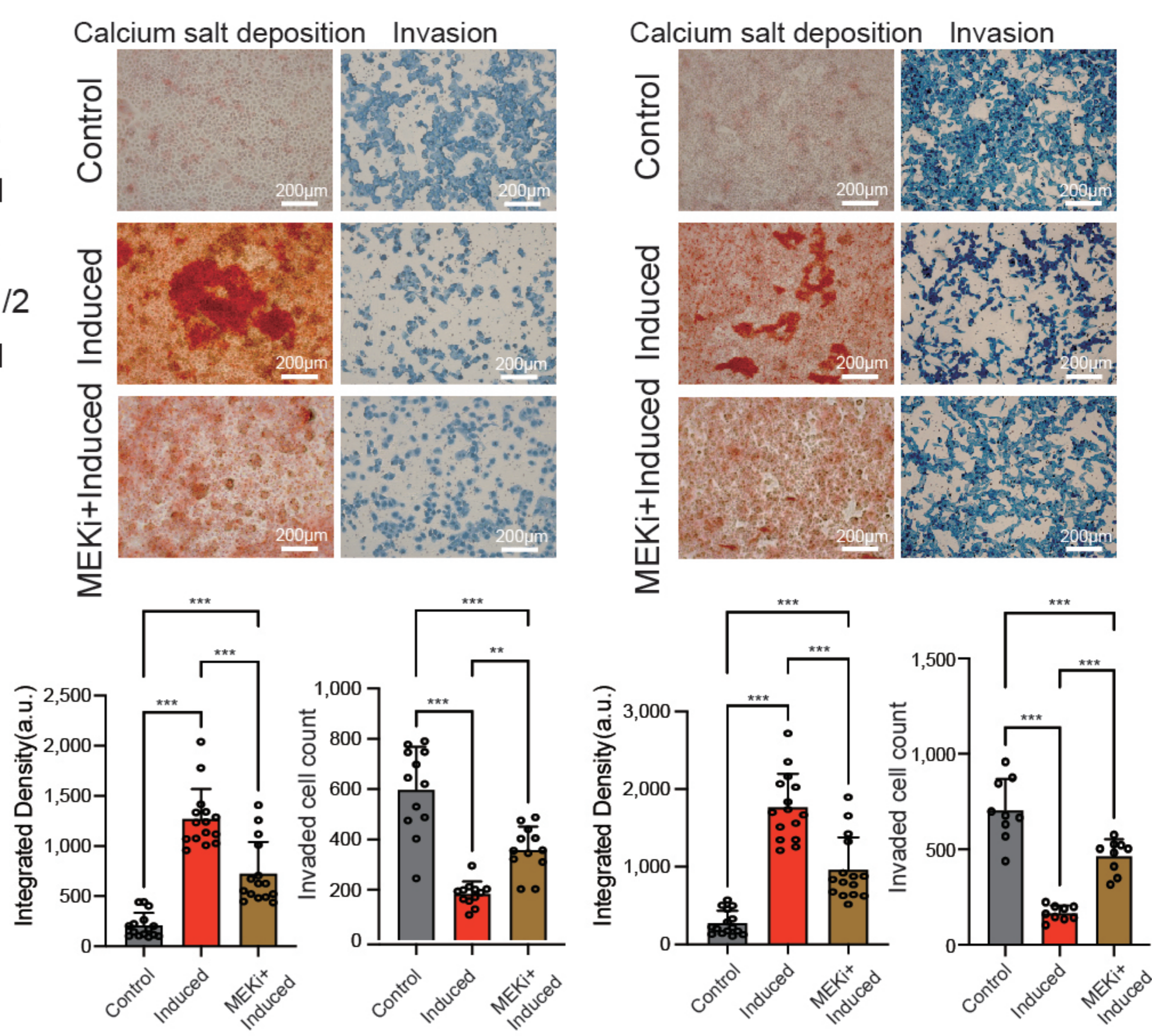

Supplement: Supplementary file 1 [file ijms-27-04725-s001.zip › FigureS4.pdf]

Figure S4D

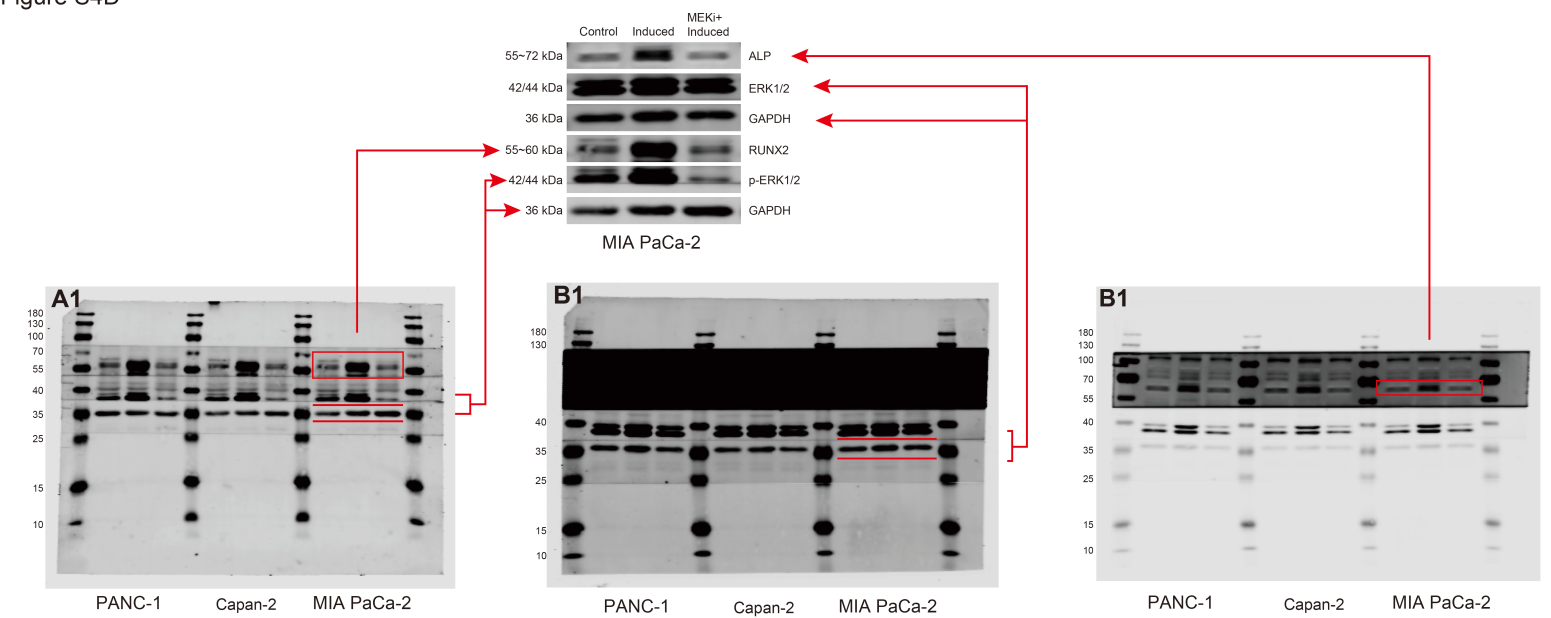

Figure 5D

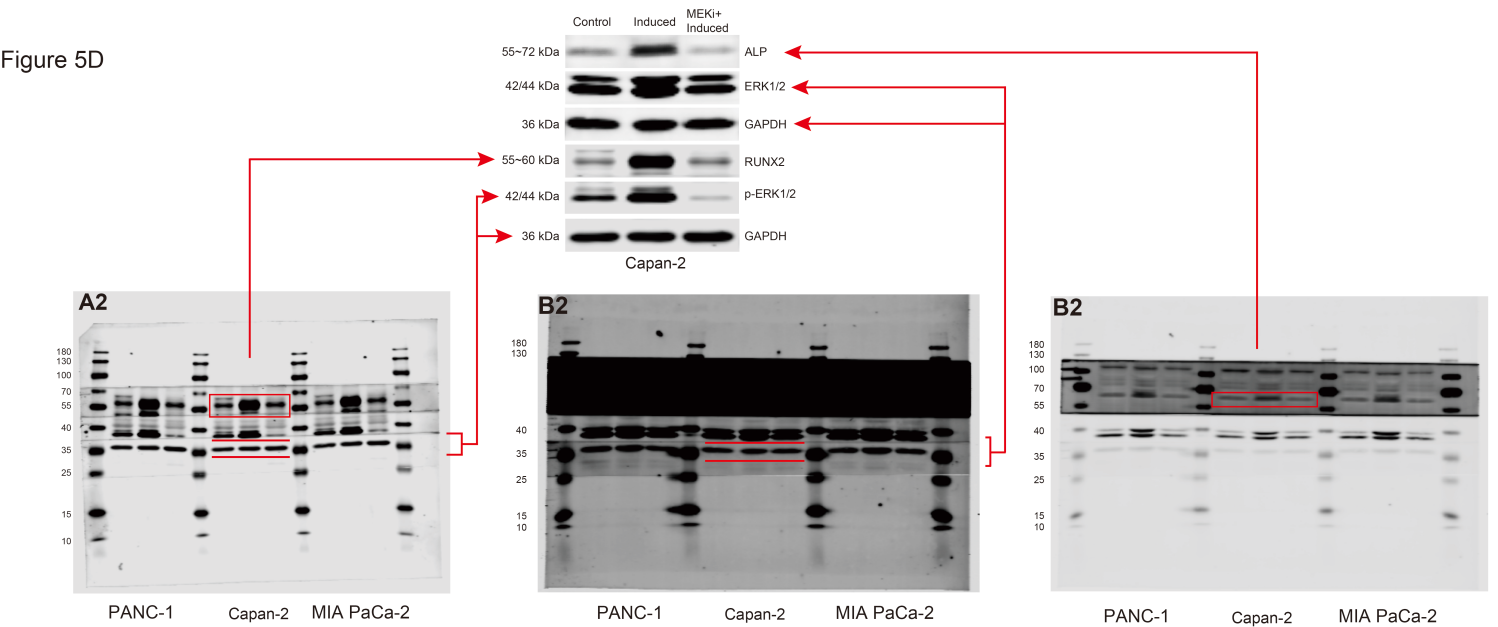

Figure S4D

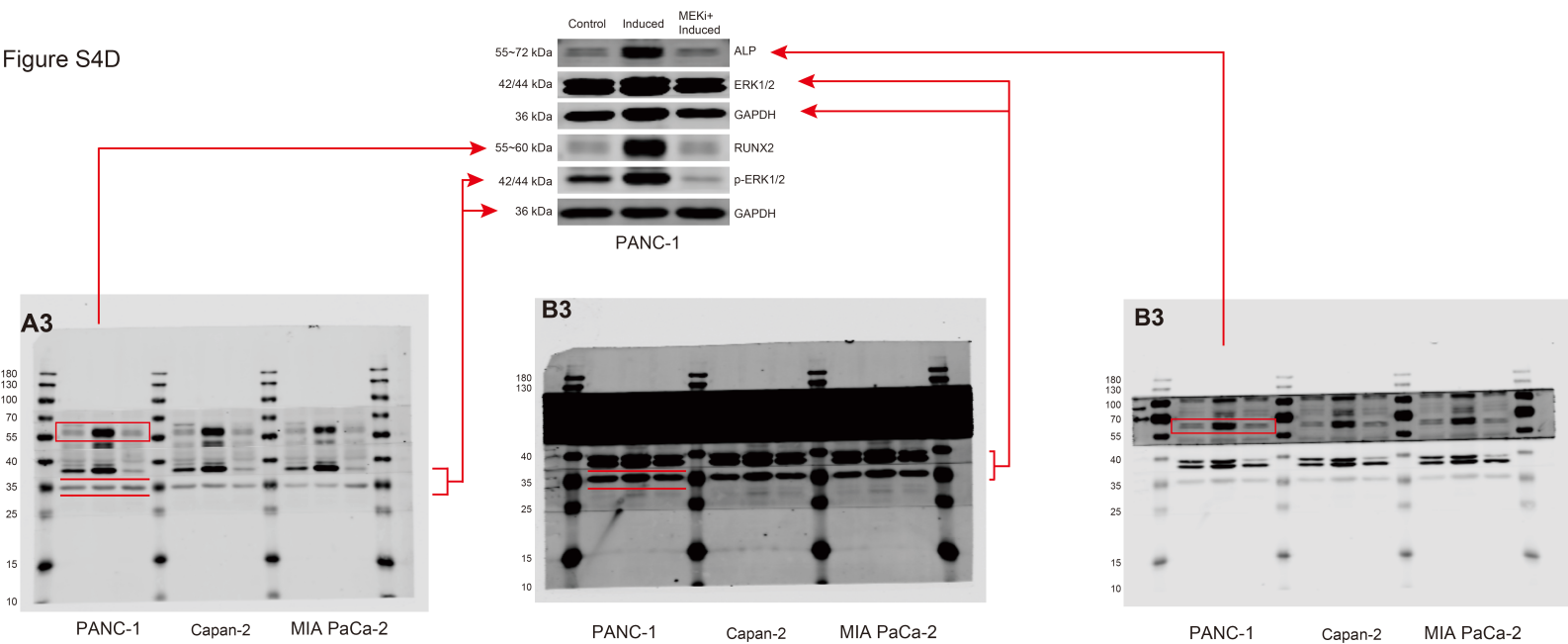

Supplement: Supplementary file 1 [file ijms-27-04725-s001.zip › Raw_WB image.pdf]

Repeat 1

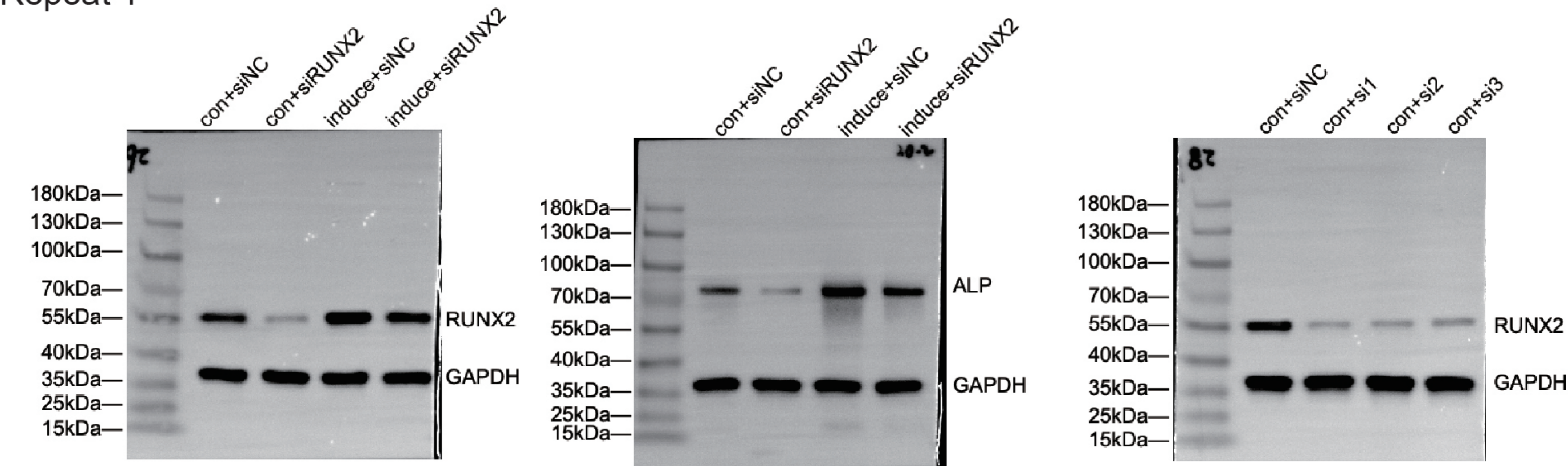

Repeat 2

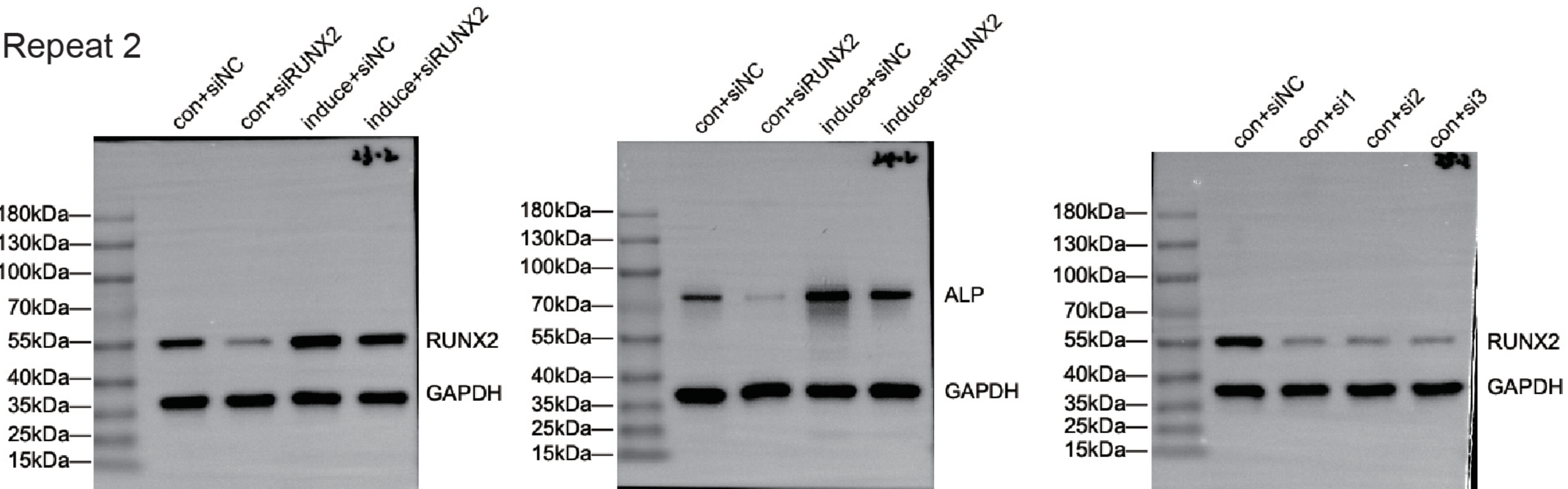

Repeat 3

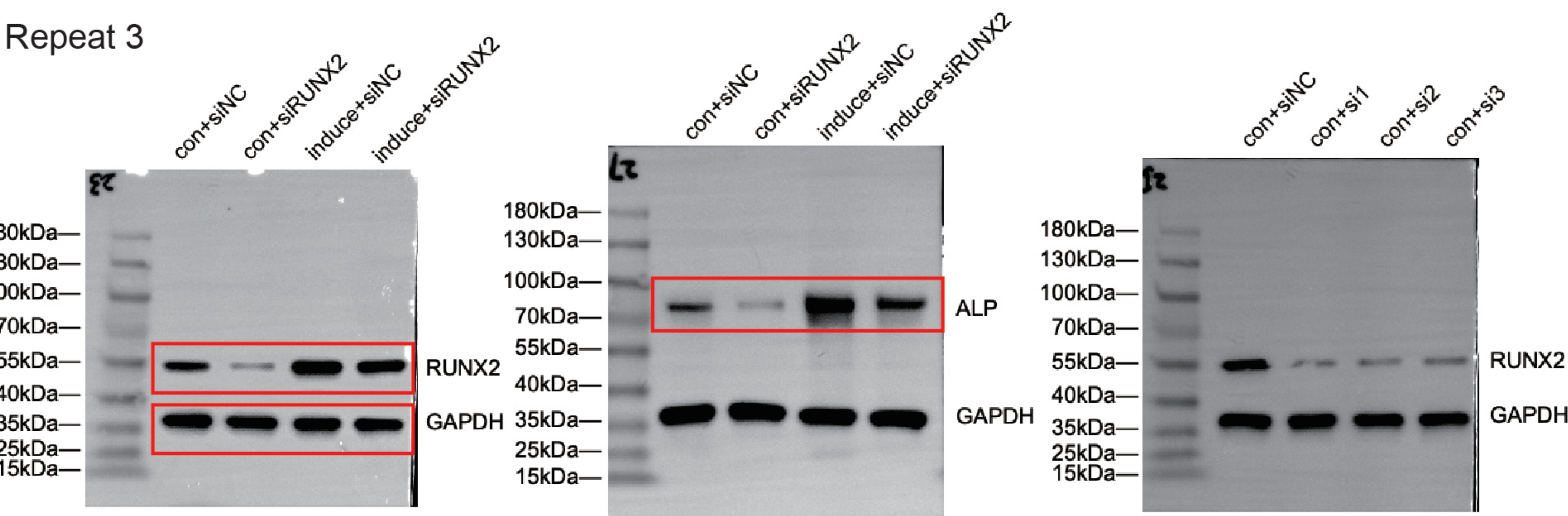

Figure 6A

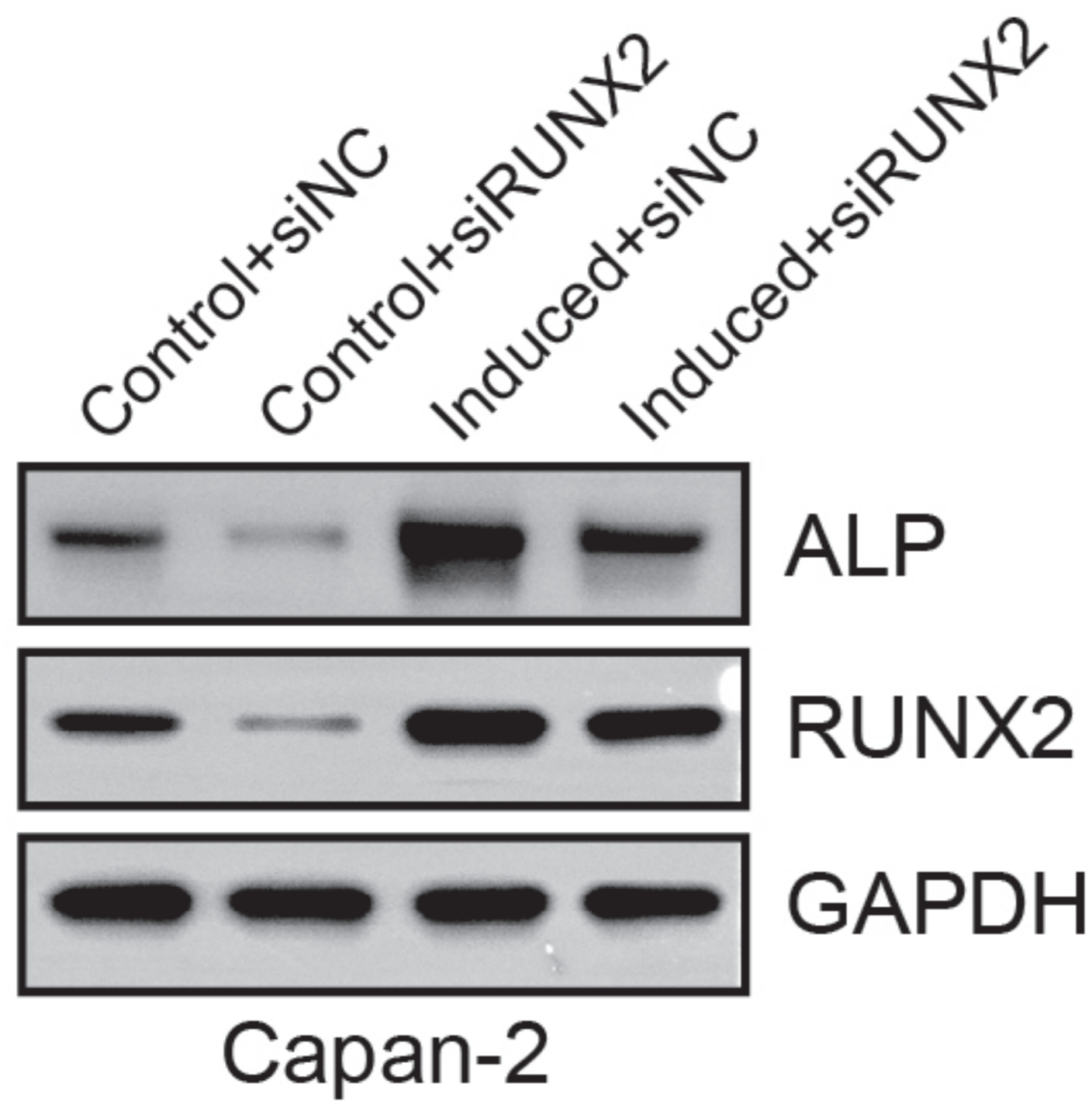

Supplement: Supplementary file 1 [file ijms-27-04725-s001.zip › Raw_wb.pdf]
